# Supplementary material for: Uncovering hidden network architecture from spiking activities using an exact statistical input-output relation of neurons
Source: Commun Biol. 2023 Feb 15;6:169. doi: 10.1038/s42003-023-04511-z (PMC9932086; doi:10.1038/s42003-023-04511-z)
Supplement: Supplementary file 2 — Supplementary Information [file 42003_2023_4511_MOESM2_ESM.pdf]

Supplementary Information

Uncovering Hidden Network Architecture from Spiking Activities Using an Exact Statistical Input-Output Relation of Neurons

Safura Rashid Shomali[1,*], Seyyed Nader Rasuli[2,3], Majid Nili Ahmadabadi[4], Hideaki Shimazaki[5,6*]

1 School of Cognitive Sciences, Institute for Research in Fundamental Sciences (IPM), Tehran 19395-5746, Iran

2 School of Physics, Institute for Research in Fundamental Sciences (IPM), Tehran 19395-5531, Iran

3 Department of Physics, University of Guilan, Rasht 41335-1914, Iran

4 Control and Intelligent Processing Center of Excellence, School of Electrical and Computer Engineering, College of Engineering, University of Tehran, Tehran 14395-515, Iran

5 Graduate School of Informatics, Kyoto University, Kyoto, Japan

6 Center for Human Nature, Artificial Intelligence, and Neuroscience (CHAIN), Hokkaido University, Hokkaido, Japan

* Corresponding authors: safura@ipm.ir, h.shimazaki@i.kyoto-u.ac.jp

Supplementary Note 1: Comparing the analytical interactions of LIF neurons with simulated results

The mixture model of the population activity builds on several assumptions that may not be valid for real or simulated activity of neurons. The first discrepancy is that, when the signaling input hits the postsynaptic voltage trajectory, it might not result in an action potential within the bin at which the signal arrives. In this case, the spontaneous rate of the subsequent bins is not the unperturbed voltage distribution that the theory assumes. The second discrepancy is that, after the signaling input arrives, sometimes, the action potential is not generated until a second signal arrives. In this case, the effect of the first signaling input is carried over to the postsynaptic voltage trajectory. When the second signaling input hits the perturbed postsynaptic voltage, the spiking probability after the signal differs from the one that the theory assumes. These effects, which we collectively call the carry-over effect, affect the spontaneous spiking probability, $F_0(\Delta)$, and the spiking probability after signal arrival, $F_A(\Delta)$. Additionally, the theory assumes only one signaling input within a bin while several signaling inputs could happen within a bin for, e.g., random signaling inputs. This could also cause discrepancies in the above

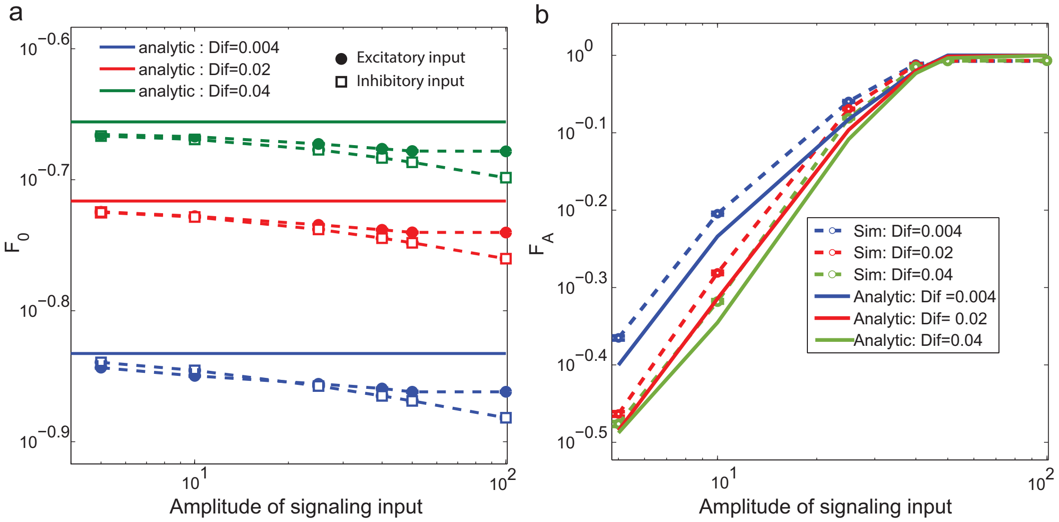

Supplementary Fig 1. Carry-over effect for spontaneous spiking probability and spiking probability after signal arrival. (**a**) The spontaneous spiking probability from the analytical result (solid lines) and simulation (dashed lines) as a function of amplitude of signaling input that the neuron received on top of noisy inputs. The spontaneous spiking probability was simulated for three different levels of scaled diffusion (Dif $= D/(\tau_\mathrm{m} V_\theta^2$, color code) for the excitatory signaling input (circles, mean $\pm$2SD) and the inhibitory signaling input (squares, mean $\pm$2SD). (**b**) Spiking probability after arrival of an excitatory signal as a function of signal amplitude (strength): analytic results (solid lines) and simulation (circles: mean $\pm$2SD, dashed lines). Each trial consisted of 10^8 steps with a step size of 0.1 ms, and 500 trials were run so as to have a small standard deviation. The parameters are: $\Delta = 5$ ms, $\lambda = 5$ Hz, $\tau_\mathrm{m} = 10$ ms, $V_\theta = 5$ mV.

probabilities. Here, we performed simulations to examine the discrepancies between the theory and simulation caused by these factors.

We traced the spontaneous spiking probability ($F_0(\Delta)$) of the LIF postsynaptic neuron by using MATLAB code when the neuron received 5 Hz Poissonian signaling input on top of noisy Gaussian inputs (mean and variance) near the threshold voltage. We used a 5 ms time window to represent the spiking activity and counted the number of bins in which the postsynaptic neuron generated a spike, but the signal did not occur. We divided it by the total number of bins lacking the signal and calculated the spiking probability. The neuron received signaling input at 5 Hz on top of noisy Gaussian inputs, balanced near the voltage near the threshold regime. The membrane time constant was $\tau_m = 10$ ms, and the threshold voltage was $V_\theta = 5\,\mathrm{mV}$. The simulations ran for duration 10^8 steps with step size 0.1 ms. We repeated the simulation 500 times. Supplementary

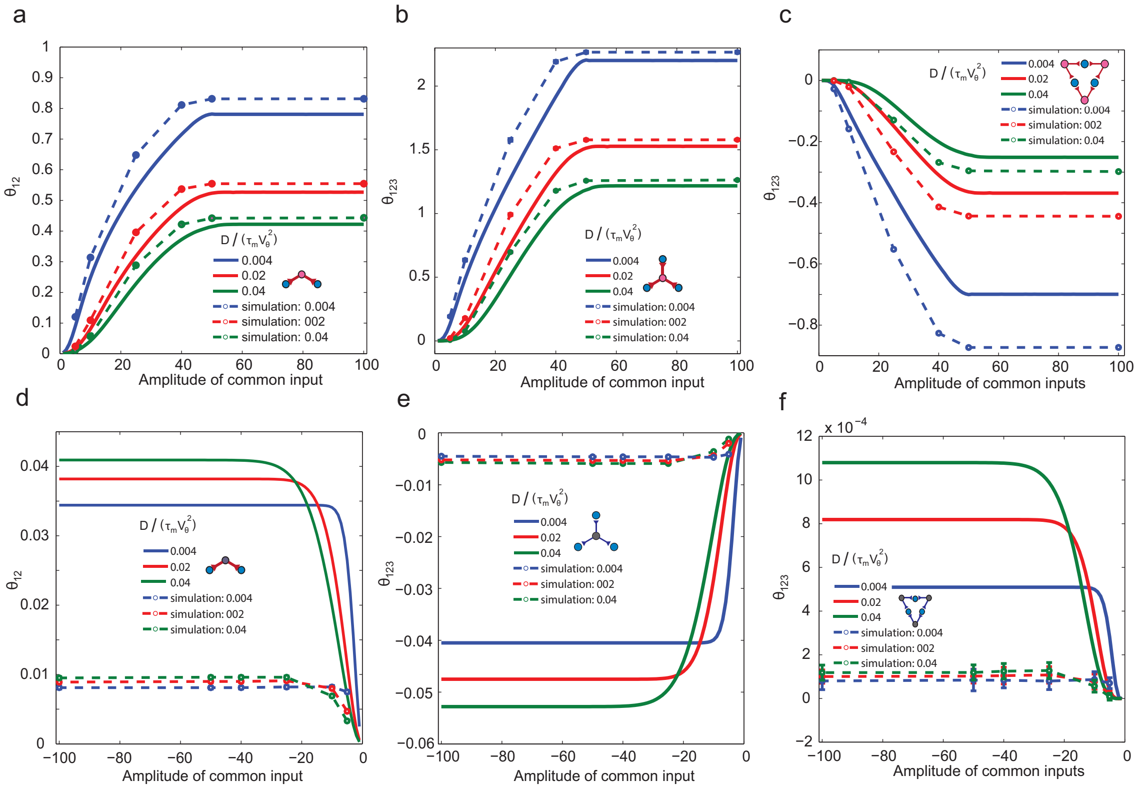

Supplementary Fig 2. Comparison of analytical and simulated pairwise and triple-wise interactions of LIF neurons receiving shared inputs on top of noisy background inputs. (**a**) Pairwise interaction between two neurons receiving common excitatory input for three levels of diffusion coefficient. Solid lines are analytical results (from Fig. 2c) and filled circles are simulated results (mean ±2 SD, SD = 0.002) for $A/(\tau_m V_\theta) = 0.1, 0.2, 0.5, 1, 2$. Three levels of background noise are indicated in blue, red and green. (**b**, **c**) Triple-wise interactions as a function of common inputs amplitude in excitatory-to-trio (b) and excitatory-to-pairs (c) motifs. Simulation results for three different noise levels (filled circles, mean ±2 SD, SD = 0.001) are compared with analytical results (solid lines, from Fig. 4c). (**d**) Simulated pairwise interactions between two neurons receiving common inhibitory input for three levels of diffusion coefficient (filled circles, mean ±2 SD, SD= 0.001). (**e**, **f**) Triple-wise interactions as a function of the amplitude of common inputs in inhibitory-to-trio (e) and inhibitory-to-pairs (f) motifs (filled circles, mean ±2 SD, SD= 0.0001, 0.00003) and analytic results (solid lines).

Fig. 1a shows the spiking probability in the bins lacking the signaling input from the simulation study with three levels of scaled diffusion coefficient ($D/\tau_{\mathrm{m}} V_\theta^2 = 0.004, 0.02, 0.04$) as a function of the strength of the signaling input (circles and squares with dashed lines). The solid lines show the analytical spontaneous spiking probability (spiking probability without signaling input). The spiking probability in the bins lacking the signaling input is decreased from the analytic spontaneous spiking probability of a neuron (solid line). This deviation is stronger when the signaling inputs' amplitude is larger. We also calculated the spiking probability after the signal

arrival, $F_A(\Delta)$. We counted the number of spikes of a postsynaptic neuron in the bins where the signal arrived and divided it by the total number of bins during which the signal occurred. Supplementary Fig. 1b shows $F_A(\Delta)$ increases for some values of excitatory signaling input.

We simulated two and three LIF neurons in different motifs to compare the pairwise and triple-wise interactions determined from the simulation with analytic results (Supplementary Fig. 2). For the pairwise and triple-wise interactions between two and three neurons for excitatory shared inputs (Supplementary Fig.2, top row), the analytical solutions (Fig. 2c and Fig. 4c) capture the increasing and saturating trend of interactions as a function of the signal amplitude. There are, however, some discrepancies between the analytical and simulation results. They are caused by the theoretical mixture model that does not consider the carry-over effect (Supplementary Fig. 1). In the simulation, we expected the spiking probability in bins lacking the signal to decrease, and the spiking probability after the signal arrival, to increase due to the carry-over effect. This effect would lead to stronger pairwise and triple-wise interactions than in the analytic results for excitatory shared inputs. We performed the same analysis on the inhibitory signaling inputs, where we observed more significant discrepancies due to the carry-over effect (Supplementary Fig.2, bottom row). However, we should note that the magnitude of the interactions caused by the inhibitory signaling inputs is much smaller than that of the excitatory signaling inputs. Further, the analytical method overestimates the interactions. Therefore, our conclusions in this study are not affected by these discrepancies.

Supplementary Note 2: Why the information-geometric measure is suitable for quantifying pairwise and higher-order interactions

We analyzed the pairwise and higher-order (i.e., triple-wise) statistical dependency among two and three neurons by using an information-geometric measure obtained from the neurons' interactions which are expressed in the form of the exponential family distribution (Eqs. 14 and 16)[37,62,64]. Here, we explain why we employed this measure instead of the alternatives.

For two neurons (Eq. 14), the information-geometric measure θ_{12} of the pairwise interaction expresses their dependency, similarly to the Pearson's correlation coefficients computed for the binary patterns. Although zero values in both measures indicate that the two neurons are uncorrelated, their mathematical expressions are different. The information-geometric measure of the pairwise interaction can take an unconstrained real value whereas the Pearson's correlation coefficients are bounded in -1 and 1.

It is known that information-geometric measures are statistically more suitable for extracting pairwise and higher-order dependencies of the binary representation of neural activity[62,64], compared with conventional measures such as Pearson's correlations, pairwise and triple-wise cross-correlations[4], cumulants[5], and mutual information[58,62,64]. For example, in the two-neuron system, the estimation of θ_{12} from the data is not affected by the estimation of the activity rates of individual neurons. This property does not hold for the other measures such as the classical Pearson's correlation coefficient[64]. Namely, the simultaneous estimation of the firing rates and the neurons' dependency measured by the Pearson's correlation coefficients are correlated. On the contrary, the information-geometric measure of the correlation θ_{12} is independent of the estimated firing rates of neurons. In this sense, it quantifies the genuine interaction that we cannot obtain from the activities of individual neurons: it can be inferred only if we simultaneously observe the two neurons. In information geometry, this property is known as orthogonality of the interaction to the activity rates of neurons.

The interactions among the three neurons (triple-wise interaction, θ_{123}) are obtained from Eq. 16. Similar to the information-geometric measure of the pairwise interactions, these triple-wise interactions estimated from the spike data are not correlated with the lower-order statistics, namely the activity rates of individual neurons and the joint activity rates of two neurons. It thus quantifies a genuine triple-wise activity of the three neurons that can be inferred only if we observe the three neurons simultaneously.

The meaning of the non-zero triple-wise interaction would become clear if we compare it with the neural activity that expresses no triple-wise interaction. Here, suppose the triple-wise interaction is fixed at zero. In that case, the model corresponds to the maximum entropy model, which is the most unstructured model when the individual firing rates and joint firing rates of pairs of neurons (equivalently, pairwise correlations) are given. Such a model produces joint activities or inactivity of all three neurons (i.e., pattern '111' or '000') with non-zero probability, but these outcomes occur as a chance coincidence given the neurons' firing rates and pairwise correlations. The non-zero information-geometric measure of the triple-wise interaction θ_{123} precisely measures the deviation of the joint activity or inactivity of the three neurons from this expected level. If it is positive, the three neurons are jointly active more frequently than the chance level expected from their activity rates and pairwise correlations. If it is negative, the neurons are simultaneously silent more frequently than the chance level.

A comparison of the other methods, such as covariance and mutual information (or Kullback-Leibler (KL) divergence), with the information-geometric measure is given in Amari[64]. The covariance method depends on firing rates, while the KL-divergence cannot reveal the sign of the correlation. Ohiorhenuan and Victor compared the KL divergence and the information-geometric measure, and their results pointed to the superiority of the information-geometric measure in practice[58]. Information-geometric measure can distinguish whether the interactions are more or less synchronous compared with that of chance level, while the KL divergence can measure only the magnitude. The information-geometric measure is also easier to calculate the confidence bound, and it was reported that it is robust to spike-sorting errors[58]. In summary, although information-geometric measure requires the data to be binarized, its statistical and practical advantages make it suitable for measuring the dependency of a neural population.

Supplementary Note 3: High-amplitude boundary for pairwise and triple-wise interactions induced by strong common excitatory and inhibitory inputs

Here, we investigated the case of an extreme strong shared signaling input and how it affects the pairwise and triple-wise interactions. This analysis helped us to find the high-amplitude boundaries for motifs in Fig. 6a. The first boundary line in the θ_{123} versus θ_{12} interaction plane for each motif arises from the high-amplitude limit (i.e., $F_A(\Delta) \to 1$ for common excitatory inputs and $F_A(\Delta) \to 0$ for common inhibitory inputs), while the spontaneous spiking probability $F_0(\Delta)$ changes within $[0, 1]$. These boundaries are shown in Fig. 6a as solid gray lines. Below, we explain, for each motif, how we found the relations for high-amplitude boundaries. First we consider pairwise interactions; then we will extend the formalism to triple-wise interactions for common excitatory and inhibitory inputs in star and (symmetric/asymmetric) triangle architectures.

Supplementary Note 3-a: Pairwise interaction for high-amplitude common input

Combining Eq. 15, Eq. 1, and Eq. 2, the pairwise interaction reads:

$$\begin{aligned}\theta_{12} &= \log \frac{(bF_A(\Delta)^2 + (1-b)F_0(\Delta)^2)(b(1-F_A(\Delta))^2 + (1-b)(1-F_0(\Delta))^2)}{(bF_A(\Delta)(1-F_A(\Delta)) + (1-b)F_0(\Delta)(1-F_0(\Delta)))^2} \\ &= \log\left[1 + \frac{b(1-b)(F_A(\Delta)-F_0(\Delta))^2}{\{bF_A(\Delta)(1-F_A(\Delta)) + (1-b)F_0(\Delta)(1-F_0(\Delta))\}^2}\right],\end{aligned} \tag{S.1}$$

where $b = \lambda\Delta$. This equation indicates that the common input induces a positive interaction for both excitatory and inhibitory connections. If the postsynaptic neuron receives an extremely strong inhibitory signal, the chance of its spiking in a short time window, Δ, immediately diminishes after signal arrival: $F_A(\Delta) \simeq 0$. This reduces Eq. S.1 to

$$\theta_{12} = \log(1 + \frac{a}{\{1-F_0(\Delta)\}^2}), \tag{S.2}$$

where $a = b/(1-b) = \lambda\Delta/(1-\lambda\Delta)$. Conversely, for an extremely strong excitatory signal, the chance of spiking in the time window, Δ, is almost 1 immediately after signal arrival; hence $F_A(\Delta) \simeq 1$. The pairwise interaction then simplifies to:

$$\theta_{12} = \log(1 + \frac{a}{F_0(\Delta)^2}). \tag{S.3}$$

Comparing Eq. S.2 and Eq. S.3, we can see that $1 - F_0(\Delta)$ simply replaces $F_0(\Delta)$. In Eq. S.3, for excitatory signaling input and with $a > 0$, we obtain a large θ_{12} when $F_0(\Delta) \ll 1$. In fact, θ_{12} indefinitely increases as $F_0(\Delta) \to 0^+$. On the other hand, for strong inhibitory signals in Eq. S.2, this criteria changes to $1 - F_0(\Delta) \ll 1$; which means we need $F_0(\Delta) \lesssim 1$ to obtain a high θ_{12}. Consequently, in the low-activity regime, i.e., $F_0(\Delta) \ll 1$, strong excitatory signals produce large θ_{12}; whereas in the high-activity regime, i.e., $F_0(\Delta) \lesssim 1$, strong inhibitory inputs produce large pairwise interactions. The only assumption in the above reasoning is the low firing rate of the presynaptic signaling input: $\lambda\Delta \ll 1$. More accurately, the Poissonian probability of one

or more signals arriving in a time window, Δ, as long as $\lambda\Delta \leq 1$, is $b = 1 - \exp(-\lambda\Delta)$, which simplifies to $b \simeq \lambda\Delta$ in the low firing rate limit of the signaling input. Accordingly, the prefactor, a, is in general, $a = \{1 - \exp(-\lambda\Delta)\}/\exp(-\lambda\Delta)$ which simplifies to $a \simeq \lambda\Delta/(1 - \lambda\Delta)$ in the low input-rate limit. However, this correction does not affect the overall result of our analysis.

Supplementary Fig. 3a shows how the pairwise interaction depends on the spontaneous spiking rate and the firing rate of the signaling input in a small time window, Δ for inhibitory (left panel) and excitatory inputs (right panel). As the firing rate of the signaling input increases (we still have $\lambda\Delta \leq 1$), the amount of interaction induced by both excitatory and inhibitory inputs increases as well. However, increasing the spontaneous spiking probability of $F_0(\Delta)$, causes the interaction induced by the excitatory-to-pairs motif to decrease while that of inhibitory-to-pairs motif to increase.

Supplementary Note 3-b: Triple-wise interaction for high-amplitude common input

Here, we extend the analysis to triple-wise interaction. First, we consider the case of strong inhibitory-to-trio motif. Combining Eq. 16, Eq. 17, and Eq. 4 gives:

$$\theta_{123} = \log \frac{P(1,1,1)P(1,0,0)^3}{P(0,0,0)P(1,1,0)^3}, \tag{S.4}$$

where

$$P(1,1,1) = bF_A(\Delta)^3 + (1-b)F_0(\Delta)^3, \tag{S.5a}$$
$$P(0,0,0) = b\{1 - F_A(\Delta)\}^3 + (1-b)\{1 - F_0(\Delta)\}^3, \tag{S.5b}$$
$$P(1,0,0) = bF_A(\Delta)\{1 - F_A(\Delta)\}^2 + (1-b)F_0(\Delta)\{1 - F_0(\Delta)\}^2, \tag{S.5c}$$
$$P(1,1,0) = bF_A(\Delta)^2\{1 - F_A(\Delta)\} + (1-b)F_0(\Delta)^2\{1 - F_0(\Delta)\}, \tag{S.5d}$$

and $b = 1 - \exp(\lambda\Delta)$, which simplifies to $b \simeq \lambda\Delta$ for the low signaling input rates. Here, we have considered the aforementioned symmetry in the inhibitory-to-trio motif, which enforces that $P(0,1,1) = P(1,0,1) = P(1,1,0)$, etc. In the strong inhibition limit, $F_A(\Delta) \simeq 0$, for small time window, $\Delta = 5 - 10\,\text{ms}$. This drastically simplifies θ_{123} to

$$\theta_{123} = -\log(1 + \frac{a}{(1 - F_0(\Delta))^3}). \tag{S.6}$$

Here, $a = \{1 - \exp(-\lambda\Delta)\}/\exp(-\lambda\Delta)$, which is approximately $a = \lambda\Delta/(1 - \lambda\Delta)$ whenever $\lambda\Delta \ll 1$. For the strong excitatory-to-trio motif, the triple-wise interaction becomes

$$\theta_{123} = \log(1 + \frac{a}{F_0(\Delta)^3}). \tag{S.7}$$

Similarly, we can consider the triple-wise interaction for excitatory-to-pairs motif, though it is more complicated compared with the inhibitory-to-trio motif. Using Eq. 16, Eq. 17 and Eq. 18, the triple-wise interaction can be written as

$$\theta_{123} = \log(\frac{P(1,1,1)P(1,0,0)^3}{P(0,0,0)P(1,1,0)^3}), \tag{S.8}$$

where

$$P(1,1,1) = (1-b)^3 F_0(\Delta)^3 + 3b(1-b)^2 F_A(\Delta)^2 F_0(\Delta) \\ + 3b^2(1-b) F_A(\Delta)^2 F_{2A}(\Delta) + b^3 F_{2A}(\Delta)^3, \tag{S.9a}$$

$$P(0,0,0) = (1-b)^3 \{1 - F_0(\Delta)\}^3 + 3b(1-b)^2 \{1 - F_A(\Delta)\}^2 \{1 - F_0(\Delta)\} \\ + 3b^2(1-b)\{1 - F_A(\Delta)\}^2 \{1 - F_{2A}(\Delta)\} + b^3 \{1 - F_{2A}(\Delta)\}^3, \tag{S.9b}$$

$$P(1,0,0) = (1-b)^3 F_0(\Delta)\{1 - F_0(\Delta)\}^2 + b^3 F_{2A}(\Delta)\{1 - F_{2A}(\Delta)\}^2 \\ + b(1-b)^2 \{2F_A(\Delta)\{1 - F_A(\Delta)\}\{1 - F_0(\Delta)\} + F_0(\Delta)\{1 - F_A(\Delta)\}^2\} \\ + b^2(1-b)\{2F_A(\Delta)\{1 - F_A(\Delta)\}\{1 - F_{2A}(\Delta)\} + F_{2A}(\Delta)\{1 - F_A(\Delta)\}^2\}, \tag{S.9c}$$

$$P(1,1,0) = (1-b)^3 F_0(\Delta)^2 \{1 - F_0(\Delta)\} + b^3 F_{2A}(\Delta)^2 \{1 - F_{2A}(\Delta)\} \\ + b(1-b)^2 \{2F_0(\Delta)\, F_A(\Delta)\{1 - F_A(\Delta)\} + F_A(\Delta)^2 \{1 - F_0(\Delta)\}\} \\ + b^2(1-b)\{2F_A(\Delta) F_{2A}(\Delta)\{1 - F_A(\Delta)\} + F_A(\Delta)^2 \{1 - F_{2A}(\Delta)\}\}. \tag{S.9d}$$

For an extremely strong excitatory-to-pairs motif, we have $F_A(\Delta) \simeq 1$ and $F_{2A}(\Delta) \simeq 1$; thus,

$$\theta_{123} = -\log\left((1 + \frac{a}{F_0(\Delta)^2})^3 \times \frac{1}{1 + 3a/F_0(\Delta)^2 + 3a^2/F_0(\Delta)^3 + a^3/F_0(\Delta)^3}\right), \tag{S.10}$$

where $a = \{1 - \exp(-\lambda\Delta)\}/\exp(-\lambda\Delta)$, as before. Moreover, we can show that the triple-wise interaction, for the case of strong inhibitory-to-pairs motif out of three neurons ($F_A(\Delta) \simeq 0$), is

$$\theta_{123} = \log\left((1 + \frac{a}{(1 - F_0(\Delta))^2})^3 \times \frac{1}{1 + 3a/(1 - F_0(\Delta))^2 + 3a^2/(1 - F_0(\Delta))^3 + a^3/(1 - F_0(\Delta))^3}\right), \tag{S.11}$$

For the asymmetric excitatory-to-pairs motif (two common inputs instead of three: neuron 1 receives from two common inputs and neuron 2 and 3 each receive from one common input, Fig. 3e), the triple-wise interaction is

$$\theta_{123} = \log\left(\frac{P(1,1,1)P(1,0,0)P(1,0,1)P(0,0,1)}{P(0,0,0)P(1,1,0)P(1,0,1)P(0,1,1)}\right), \tag{S.12}$$

while the pattern probabilities S.9 are:

$$P(1,1,1) = (1-b)^2F_0(\Delta)^3 + 2b(1-b)F_A(\Delta)^2F_0(\Delta) + b^2F_A(\Delta)^2F_{2A}(\Delta), \tag{S.13a}$$
$$P(0,0,0) = (1-b)^2\{1-F_0(\Delta)\}^3 + 2b(1-b)\{1-F_A(\Delta)\}^2\{1-F_0(\Delta)\} + b^2\{1-F_A(\Delta)\}^2\{1-F_{2A}(\Delta)\}, \tag{S.13b}$$
$$P(1,0,0) = (1-b)^2F_0(\Delta)\{1-F_0(\Delta)\}^2 + b^2F_{2A}(\Delta)\{1-F_A(\Delta)\}^2 + 2b(1-b)F_A(\Delta)\{1-F_A(\Delta)\}\{1-F_0(\Delta)\} \tag{S.13c}$$
$$P(0,1,0) = P(0,0,1) = (1-b)^2F_0(\Delta)\{1-F_0(\Delta)\}^2 + b^2F_A(\Delta)\{1-F_A(\Delta)\}\{1-F_{2A}(\Delta)\} + b(1-b)\big\{F_A(\Delta)\{1-F_A(\Delta)\}\{1-F_0(\Delta)\} + F_0(\Delta)\{1-F_A(\Delta)\}^2\big\} \tag{S.13d}$$
$$P(1,1,0) = P(1,0,1) = (1-b)^2F_0(\Delta)^2\{1-F_0(\Delta)\} + b^2F_{2A}(\Delta)F_A(\Delta)\{1-F_A(\Delta)\} + b(1-b)\big\{F_A(\Delta)^2\{1-F_0(\Delta)\} + F_A(\Delta)F_0(\Delta)\{1-F_A(\Delta)\}\big\} \tag{S.13e}$$
$$P(0,1,1) = (1-b)^2F_0(\Delta)^2\{1-F_0(\Delta)\} + b^2F_A(\Delta)^2\{1-F_{2A}(\Delta)\} + 2b(1-b)F_A(\Delta)F_0(\Delta)\{1-F_A(\Delta)\}. \tag{S.13f}$$

In high-amplitude limit of asymmetric excitatory-to-pairs motif ($F_A(\Delta) \simeq 1$), the triple-wise interaction becomes

$$\theta_{123} = -\log\left((1+\frac{a}{F_0(\Delta)^2})^2 \times \frac{1}{1+2a/F_0(\Delta)^2 + a^2/F_0(\Delta)^3}\right). \tag{S.14}$$

Supplementary Note 3-c: Marginalized pairwise interaction among three neurons for high-amplitude common input

The pairwise interaction, θ_{12}, for two neurons among the three (i.e., marginalized pairwise interaction) can be written using the probability of patterns for three neurons as

$$\theta_{12} = \log\left(\frac{(P(1,1,1)+P(1,1,0))(P(0,0,0)+P(0,0,1))}{(P(1,0,1)+P(1,0,0))(P(0,1,1)+P(0,1,0))}\right), \tag{S.15}$$

For the excitatory-to-trio (inhibitory-to-trio) motif, the marginal θ_{12} is simply obtained by inserting Eq. S.5 in Eq. S.15. For strong excitatory (inhibitory) input, $F_A(\Delta) \simeq 1$ ($F_A(\Delta) \simeq 0$), the result is the same as Eq. S.3 (Eq. S.2). The marginalized θ_{12} for the symmetric excitatory-to-pairs motif is calculated by inserting Eq S.9 in Eq S.15 when $F_A(\Delta) \simeq 1$ for strong excitatory inputs ($F_A(\Delta) \simeq 0$ for strong inhibitory inputs). The marginalized θ_{12} for high-amplitude signals in excitatory-to-pairs motifs is (for inhibitory-to-pairs replace $F_0(\Delta)$ with $1 - F_0(\Delta)$) as follows:

$$\theta_{12} = \log\left((1+2a/F_0(\Delta) + a/F_0(\Delta)^2 + 3a^2/F_0(\Delta)^2 + a^3/F_0(\Delta)^2)/(1+a/F_0(\Delta))^2\right). \tag{S.16}$$

For the asymmetric excitatory-to-pairs motif, as in the symmetric case, we put the relevant pattern probabilities, Eq. S.13 in Eq. S.15, while $F_A(\Delta) \simeq 1$ for strong excitatory inputs. Because this architecture is asymmetric (see Fig. 3e), just two marginalized pairwise interactions among the three are nonzero (here, $\theta_{23} = 0$). The marginalized θ_{12} for the asymmetric excitatory-to-pairs

motif in high-amplitude regime is (for the asymmetric inhibitory-to-pairs motif replace $F_0(\Delta)$ with $1 - F_0(\Delta)$):

$$\theta_{12} = \theta_{13} = \log\left((1 + a/F_0(\Delta) + a/F_0(\Delta)^2 + a^2/F_0(\Delta)^2)/(1 + a/F_0(\Delta))\right). \tag{S.17}$$

Supplementary Fig. 3b shows, for a postsynaptic neuron with low activity, i.e., $F_0(\Delta) \ll 1$, the excitatory-to-pairs motif generates strong negative triple-wise interaction (right panel); whereas for high spontaneous spiking probability, i.e., $F_0(\Delta) \simeq 1$, the inhibitory-to-trio motif produces strong interactions.

Supplementary Note 3-d: High-amplitude for very small pairwise and triple-wise interactions of excitatory/inhibitory to trio motif

In the case of giving high-amplitude excitatory inputs to three neurons ($F_A(\Delta) \to 1$), Eq. S.3 and Eq. S.7 draw the high-amplitude boundary over the whole range of $F_0(\Delta)$ in the θ_{123} versus θ_{12} plane, but a small region ($0 < \theta_{12} < \log(1 + a)$, $0 < \theta_{123} < \log(1 + a)$) remains, where the neuron operates in very high spontaneous regime, $F_0(\Delta) \to 1$. Since the excitatory input is in high-amplitude regime (($F_A(\Delta) \to 1$) and simultaneously the spontaneous rate is very high ($F_0(\Delta) \to 1$), the relations for pairwise and triple-wise interactions (Eq. S.1 and Eq. S.4) are indeterminate. In this regime, we can take the limit of the pairwise and triple-wise interactions (Eq. S.1, Eq. S.4 and Eq. S.5), by considering $F_A(\Delta) \to 1$ and $F_0(\Delta) \to 1$ simultaneously. Assuming $x_e = (1 - F_A(\Delta)/(1 - F_0(\Delta))$, the pairwise relation, Eq. S.1, is

$$\theta_{12} = \log\frac{(ax_e^2 + 1)(a + 1)}{(ax_e + 1)^2}. \tag{S.18}$$

Here, $a = b/(1 - b)$, $b = \lambda\Delta$ and x_e varies within the range $[0, 1]$. The triple-wise interaction for excitatory-to-trio motif (Eq. S.4 and Eq. S.5) in the limit of $F_A(\Delta) \to 1$ and $F_0(\Delta) \to 1$ becomes

$$\theta_{123} = \log\frac{(ax_e^2 + 1)^3(a + 1)}{(ax_e^3 + 1)(ax_e + 1)^3}. \tag{S.19}$$

In this limit, the pairwise and triple-wise interactions vary between $\log(1 + a)$ and 0, for the range of x_e within $[0, 1]$.

Similarly, for inhibitory-to-trio motif, in the high-amplitude limit ($F_A(\Delta) \to 0$), the pairwise and triple-wise interactions (Eq. S.1 and Eq. S.4) become indeterminate, when the neuron has a very low spontaneous rate ($F_0(\Delta) \to 0$): $0 < \theta_{12} < \log(1 + a)$, $-\log(1 + a) < \theta_{123} < 0$. In this limit, we simultaneously set $1 - F_A(\Delta) \to 1$ and $1 - F_0(\Delta) \to 1$. Then, the pairwise interaction in Eq. S.1 reduces to

$$\theta_{12} = \log\frac{(ax_i^2 + 1)(a + 1)}{(ax_i + 1)^2}, \tag{S.20}$$

where $x_i = F_A(\Delta)/F_0(\Delta)$ and varies within the range $[0, 1]$. The triple-wise interactions (Eq. S.4 and Eq. S.5) become:

$$\theta_{123} = \log\frac{(ax_i^3 + 1)(ax_i + 1)^3}{(ax_i^2 + 1)^3(a + 1)}. \tag{S.21}$$

The pair of pairwise and triple-wise interactions, $(\theta_{12}, \theta_{123})$ in this limit for a change of x_i within $[0, 1]$, vary from $(\log(1 + a), -\log(1 + a))$ to (0, 0).

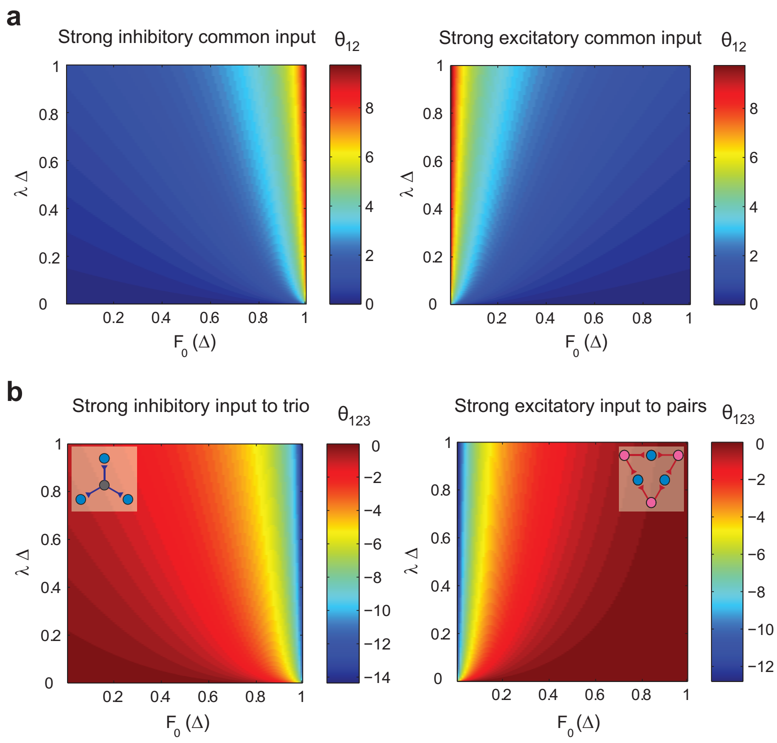

Supplementary Fig 3. Pairwise and triple-wise interactions generated by strong common excitatory and inhibitory inputs. (**a**) The pairwise interactions of two neurons generated by strong common inhibitory (left) and excitatory (right) inputs as a function of the spontaneous spike rate of a postsynaptic neuron $F_0(\Delta)$ and input rate $\lambda\Delta$. Common Inhibitory inputs generate strong pairwise interactions if the spontaneous rate of the postsynaptic neuron is high (i.e., $F_0(\Delta) \simeq 1$), while common excitatory inputs generate strong interactions in the low spontaneous rate regime, $F_0(\Delta) \ll 1$. (**b**) The triple-wise interactions among three neurons generated by strong common inputs in the inhibitory-to-trio (left) and excitatory-to-pairs (right) motifs. The same tendency found in the pairwise interactions applies to the triple-wise interaction. The excitatory-to-pairs motif generates strong triple-wise interactions in the low spontaneous rate regime, but the inhibitory-to-trio motif cannot induce such strong interactions in this regime.

Supplementary Note 4: Defining the analytical low (high) spontaneous rate boundaries for the excitatory (inhibitory) to trios/pairs in θ_{123} versus θ_{12} plane

Here we describe in brief how to determine the low (high) spontaneous rate boundary for excitatory (inhibitory) input based motifs in the θ_{123} versus θ_{12} plane. One can easily find out how the spontaneous postsynaptic neuron's firing rate relates to the level of background synaptic activities from a stationary distribution of voltage trajectories for the LIF model neuron[8,100]: i.e., (Appendix V in Shomali *et al.*[36])

$$P_s(V) = \frac{\mu\tau_{\rm m}^2}{D}\int_V^{\bar{I}} \Theta(V_s - V_r)\exp\big(\frac{-\tau_{\rm m}}{2D}(V^2 - V_s^2 - 2\bar{I}(V - V_s))\big)dV_s, \tag{S.22}$$

where $\Theta(V)$ is the Heaviside step function: $\Theta(V) = 1$ for $V > 0$ and otherwise $\Theta(V) = 0$. μ is the mean firing rate: $\mu = (\int_0^\infty sJ_0(s)\,ds)^{-1}$ and V_r is the resting voltage that we assumed to be zero. $\bar{I}$ is the mean current which is equal to V_θ for the threshold regime. Since the stationary distribution is normalized ($\int_0^\infty P_s(V)dV_s = 1$), μ can be obtained explicitly[8]:

$$\mu^{-1} = \tau_{\rm m}\sqrt{\pi}\int_{(V_r-\bar{I})\sqrt{\tau_{\rm m}/2D}}^{(V_\theta-\bar{I})\sqrt{\tau_{\rm m}/2D}} e^{y^2}(1 + {\rm erf}(y))dy, \tag{S.23}$$

where ${\rm erf}(x) = (2/\sqrt{\pi})\int_0^x \exp(-t^2)dt$. For $V_r = 0$ and $\bar{I} = V_\theta$ (the threshold regime), the firing rate becomes

$$\mu = (2\tau_{\rm m}\int_{-\sqrt{\frac{\tau_{\rm m}V_\theta^2}{2D}}}^{0}\int_{-\infty}^{x}\exp(x^2 - t^2)dt\,dx)^{-1}. \tag{S.24}$$

Using Eq. S.24, each level of background input noise (diffusion coefficient) gives us a specific spontaneous firing rate that we can use to calculate $F_0(\Delta)$. In the star architecture with common excitatory input, for fixed $F_0(\Delta)$, by changing $F_A(\Delta)$ within the valid range $[F_0(\Delta), 1]$, we determined the whole range of triple-wise and pairwise interactions for excitatory-to-trio motif (i.e., Eq. S.15 and Eq. S.4) associated with a specific spontaneous rate (green to purple dashed lines in Fig. 6a). We observed that, as the background input noise decreased, the region for excitatory-to-trio motif expands, so we used the lowest plausible spontaneous rate to determine the boundary (we used $\mu = 1$ Hz for $D = 5.5 \times 10^{-17} ms \cdot mV^2$). This gave us the spontaneous spiking probability, $F_0(\Delta) = 0.005$ (for $\Delta = 5\,$ms). By changing $F_A(\Delta)$ within the range $[F_0(\Delta), 1]$ in Eq. S.15 and Eq. S.4, we obtained the second boundary for the excitatory-to-trio motif (Fig. 6a, purple line).

For inhibitory-to-trio motif, a higher spontaneous rate shows stronger interactions. Thus, the second boundary is the limit of the high spontaneous rate. Since we are restricted to a low spontaneous rate regime (i.e., $F_0(\Delta) \leq 0.5$), we chose the background noise for $F_0(\Delta) = 0.5$, that is ($D = 190\,{\rm ms}\cdot{\rm mV}^2$ and $\mu = 100$ Hz for $\Delta = 5$ ms). Here, because of the inhibitory input, $F_A(\Delta)$ varies within the range $[0, F_0(\Delta)]$ in Eq. S.15 and Eq. S.4, but this time for the high spontaneous rate ($\mu = 100$ Hz, $F_0(\Delta) = 0.5$). The two boundaries for the inhibitory-to-trio region (Fig. 6a left, bottom) lie very close to each other so the region is narrow in the plane of triple-wise versus pairwise interactions for low spontaneous rate regime (i.e., $0 \leq F_0(\Delta) \leq 0.5$).

For inhibitory-to-pairs motif, again the second boundary is the high spontaneous rate ($D = 190\,\mathrm{ms}\cdot\mathrm{mV}^2$ and $\mu = 100$ Hz, $F_0(\Delta) = 0.5$ for $\Delta = 5$ ms). This boundary was found by simulating LIF neurons for $D = 190 ms \cdot mV^2$, to find the $F_A(\Delta)$ and $F_{2A}(\Delta)$ relation in the neuron model. Then, we calculated pairwise and triple-wise interactions by inserting the obtained relation between $F_A(\Delta)$ and $F_{2A}(\Delta)$(Eq. 13, Eq. S.15 and Eq. 17).

Finally, the second boundary for the excitatory-to-pairs motif in the low spontaneous rate regime is obtained using very low diffusion ($\mu = 1$ Hz for $D = 5.5\times10^{-17}\,\mathrm{ms}\cdot\mathrm{mV}^2$). As in the case of the inhibitory-to-pairs motif, the boundary depends on both $F_A(\Delta)$ and $F_{2A}(\Delta)$ and consequently on the neuron model. However, the LIF simulation in this very low spontaneous rate does not give reliable results ($D = 5.5\times10^{-17} ms \cdot mV^2$, $\tau_\mathrm{m} = 10ms$, $V_\theta = 5mV$). Thus, we took a different approach; we derived the postsynaptic neuron's CDF after signal arrival (i.e., $F_A(\Delta)$ and consequently $F_{2A}(\Delta)$) for the very low spontaneous rate regime:

$$F_A = \int_{V_\theta - A/\tau_m}^{V_\theta} P_s(V)dV, \tag{S.25}$$

where $P_s(v)$ is the stationary distribution (Eq. S.22). We find the instantaneous spiking just after the signal arrives, and because of very low diffusion, the neuron doesn't generate spike for a while after signal arrival. When $\frac{A}{\tau_\mathrm{m}} \le V_\theta$, the CDF is:

$$F_A = \mu\tau_\mathrm{m} \int_0^{A/\sqrt{D\tau_\mathrm{m}}} \frac{1}{u}\left(e^{-\frac{u^2}{2}} - e^{\frac{u^2}{2} - uA/\sqrt{D\tau_\mathrm{m}}}\right)du, \tag{S.26}$$

and for $\frac{A}{\tau_\mathrm{m}} > V_\theta$, it is

$$\begin{aligned} F_A = \mu\tau_\mathrm{m} &\int_0^{A/\sqrt{D\tau_\mathrm{m}} - V_\theta\sqrt{\tau_m/D}} \frac{1}{u}\left(e^{-\frac{u^2}{2}} - e^{-\frac{u^2}{2} - uV_\theta\sqrt{\tau_\mathrm{m}/D}}\right)du + \\ &\int_{A/\sqrt{D\tau_\mathrm{m}} - V_\theta\sqrt{\tau_m/D}}^{A/\sqrt{D\tau_\mathrm{m}}} \frac{1}{u}\left(e^{-\frac{u^2}{2}} - e^{\frac{u^2}{2} - uA/\sqrt{\tau_\mathrm{m}D}}\right)du. \end{aligned} \tag{S.27}$$

For a specific spontaneous firing rate of the postsynaptic neuron, by using Eq. S.26 and Eq. S.27, pairwise and triple-wise interactions are calculated (in Eq. S.15 and Eq. 17) and drawn in Fig.6a. As shown in Figure 6a, the boundary in the very low spontaneous rate regime, is determined ($\mu = 1$ Hz) for both symmetric and asymmetric excitatory-to-pairs motif(purple dashed lines in the excitatory-to-pairs region, Fig.6a). At high spontaneous rates (Fig. 10b), the boundary for the lowest spontaneous rate ($\mu = 100$ Hz) of the excitatory-to-pairs motif, is found by simulating the LIF neuron model at a specific diffusion that relates to $\mu = 100$ Hz, finding the $F_A(\Delta)$ and $F_{2A}(\Delta)$ relation, and calculating interactions from Eq. S.15 and Eq. 17. So like one boundary of inhibitory-to-pairs motif, this boundary depends on the neuron model (here LIF) in Fig. 6a. Regarding these boundaries (Supplementary Note 3, 4), we build a guide map for inferring basic motifs from neural interactions. A guide map for neural interactions is presented for postsynaptic low spontaneous rate of $\mu = 1 - 100$ Hz (Fig. 10a). For high spontaneous rate, $\mu = 100 - 1000$ Hz, the guide map changes in favor of inhibitory inputs motifs for strong interactions (Fig. 10b). Supplementary Fig. 4 shows the excitatory inputs regions inducing weak interactions between postsynaptic neurons at high spontaneous rates.

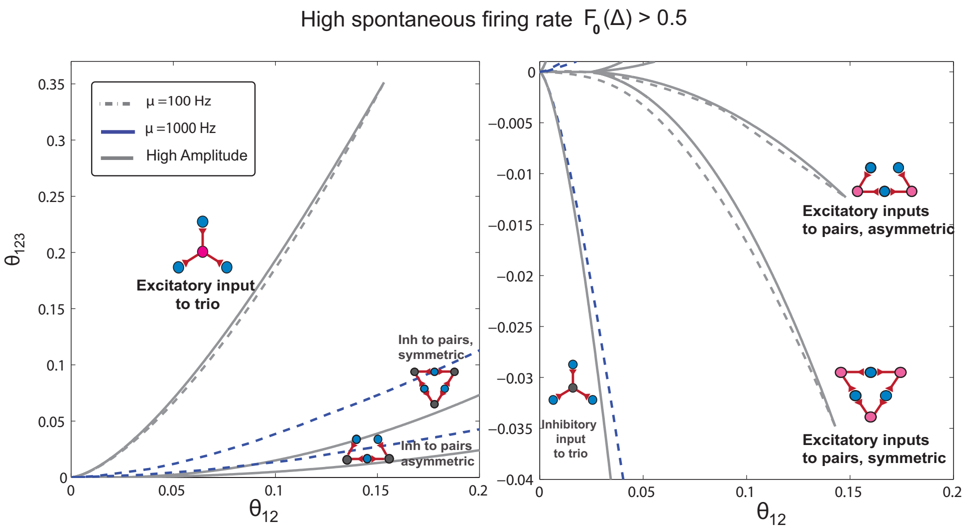

Supplementary Fig 4. Magnified views of Fig. 10b. Regions of triple-wise interaction versus pairwise interaction for star and triangular architectures with common excitatory and inhibitory inputs under high spontaneous firing rates of postsynaptic neurons $\mu = 100 - 1000$ Hz. In this regime, the excitatory inputs' motifs induce small interactions, making their regions smaller than those found at low postsynaptic spontaneous rates (Fig. 6a). The only fixed parameters are the bin size $\Delta = 5$ ms and the presynaptic rate $\lambda = 5$ Hz.

Note that the carry-over effect (Supplementary Note 1) decreases the spontaneous rate after applying the signaling input. Therefore, the results of the pairwise and triple-wise interactions (Fig. 6a) shift to lines for a lower spontaneous rate.

The dependence on bin size is depicted in Supplementary Fig. 5; it shows how different bin sizes change the regions of the guide map for spontaneous rates of $\mu = 1 - 100$ Hz. When larger bin sizes are chosen, the regions for the excitatory motifs in low spontaneous rate regime ($\mu = 1 - 100$ Hz), shrink.

Supplementary Note 4-a: How does excitatory-to-trio motif produce negative values for θ_{123}?

We observed that when the spontaneous rate of the postsynaptic neuron is less than $\mu = 5$ Hz, the triple-wise interaction in the motif of excitatory inputs given to three neurons, becomes negative for some values of common input's amplitude (Fig. 6a). We can rewrite the pattern probabilities and triple-wise interaction for excitatory inputs given to three neurons from Eq. S.4

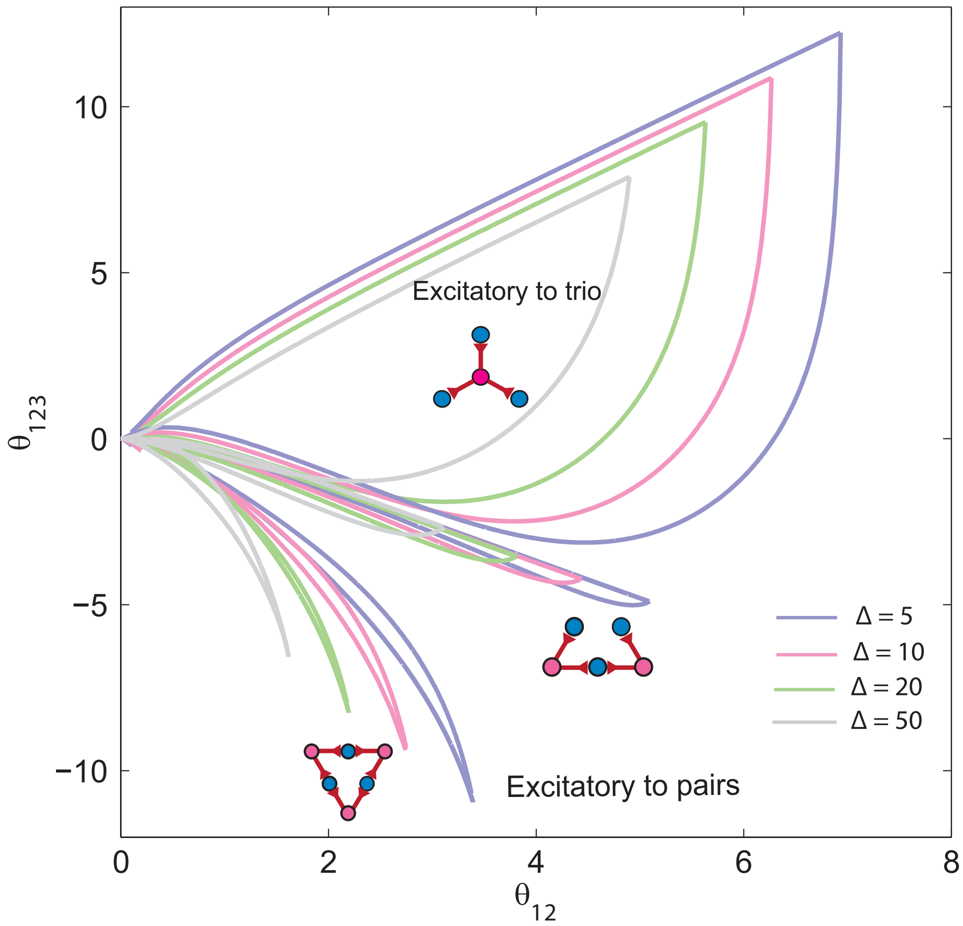

Supplementary Fig 5. Dependence of the guide map on the bin size. The map shows the dependence of the guide map on the bin size $\Delta = 5$ ms (blue), $\Delta = 10$ ms (pink), $\Delta = 20$ ms (green) and $\Delta = 50$ ms (gray) for star and triangular architectures with common excitatory inputs under low spontaneous rate regime of postsynaptic neurons $\mu = 1 - 100$ Hz. The common input rate is $\lambda = 5\,\text{Hz}$. The boundaries for the excitatory motifs shrink when larger bin sizes are chosen.

and Eq. S.5 as

$$P(1,1,1) = F_0(\Delta)^3(ax^3+1), \tag{S.28a}$$

$$P(1,1,0) = F_0(\Delta)^2(1-F_0(\Delta))(ax^2(\frac{1-xF_0(\Delta)}{1-F_0(\Delta)})+1), \tag{S.28b}$$

$$P(1,0,0) = F_0(\Delta)(1-F_0(\Delta))^2(ax(\frac{1-xF_0(\Delta)}{1-F_0(\Delta)})^2+1), \tag{S.28c}$$

$$P(0,0,0) = (1-F_0(\Delta))^3(a(\frac{1-xF_0(\Delta)}{1-F_0(\Delta)})^3+1). \tag{S.28d}$$

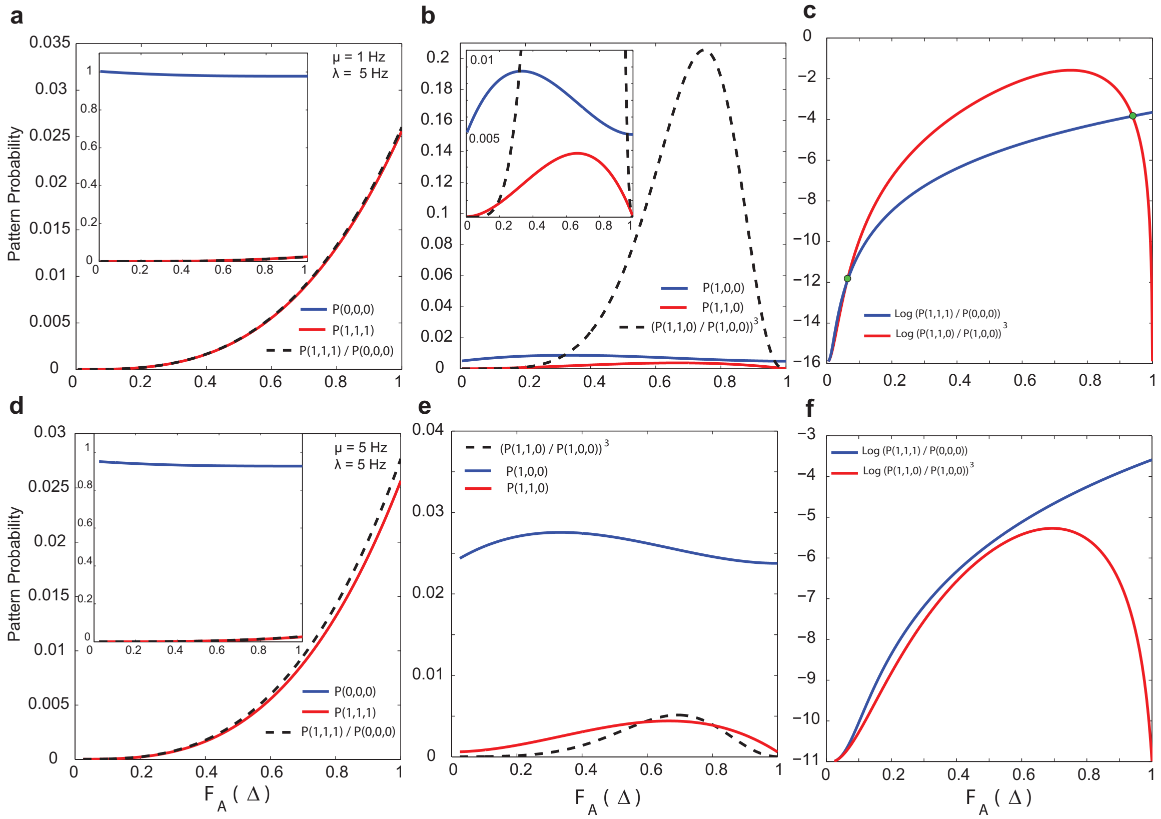

Supplementary Fig 6. Pattern probabilities for three neurons. (**a**, **b**, **c**) Pattern probabilities for neurons with spontaneous rate of 1 Hz receiving common signaling input with rate $\lambda = 5$ Hz (excitatory-to-trio motif). If φ_2^3 is bigger than φ_1, the triple-wise interaction becomes negative. In this condition, we have $\log \varphi_2^3 > \log \varphi_1$ for some ranges of amplitudes which leads to the negative triple-wise interactions (c, green dots mark the range), (**d**, **e**, **f**) The pattern probabilities when the spontaneous rate is 5 Hz. At $F_0 = 5$ Hz, there is no region in which $\log \varphi_2^3 > \log \varphi_1$ that induces negative triple-wise interactions (f, bottom right).

The triple-wise interaction is

$$\theta_{123} = \log \frac{P(1,1,1)P(1,0,0)^3}{P(0,0,0)P(1,1,0)^3} = \log\left(\varphi_1 \times \varphi_2^{-3}\right), \tag{S.29}$$

where

$$\varphi_1 = \frac{P(1,1,1)}{P(0,0,0)} = \frac{F_0(\Delta)^3}{(1-F_0(\Delta))^3} \times \frac{ax^3+1}{a(\frac{1-xF_0(\Delta)}{1-F_0(\Delta)})^3+1}, \tag{S.30}$$

and

$$\varphi_2 = \frac{P(1,1,0)}{P(1,0,0)} = \frac{F_0(\Delta)^2(1-F_0(\Delta))}{F_0(\Delta)(1-F_0(\Delta))^2} \times \frac{ax^2\frac{1-xF_0(\Delta)}{1-F_0(\Delta)}+1}{ax(\frac{1-xF_0(\Delta)}{1-F_0(\Delta)})^2+1}, \tag{S.31}$$

where $a = b/(1-b)$, $b = \lambda\Delta$ and $x = F_A(\Delta)/F_0(\Delta)$. For the excitatory-to-trio motif, x varies within the range $1 \leq x \leq 1/F_0(\Delta)$. The spontaneous rate of $\mu = 1$ Hz for a time window $\Delta = 5$ ms can generate negative triple-wise interactions for some range of common input amplitude (Supplementary Fig. 6, top panels). However the spontaneous rate of $\mu = 5$ Hz generates positive triple-wise interaction over the whole range of common input amplitude (Supplementary Fig. 6, bottom panels). The term that makes the triple-wise interaction, negative at some values of common input amplitude for low rates is φ_2: it is the fraction of pattern probability $P(1,1,0)$ over $P(1,0,0)$ (Supplementary Fig. 6, b). When φ_2^3 exceeds φ_1, Eq. S.29 gives a negative triple-wise interaction for excitatory-to-trio motif (Supplementary Fig. 6, compare top with bottom panels).

By investigation, we found that when $\eta < 0.25$, where $\eta = F_0(\Delta)(1 - F_0(\Delta))/a^{2/3}$, the triple-wise interaction becomes negative in the excitatory-to-trio motif. Under this condition, a negative triple-wise interaction occurs when the cumulative density function $F_A(\Delta)$ is in the range $F_-(\Delta) \leq F_A(\Delta) \leq F_+(\Delta)$, where $F_\pm(\Delta) = 0.5 \pm \sqrt{0.25 - \eta}$. For example, when the common input rate is $\lambda = 5$ Hz and the time window is $\Delta = 5$ ms, postsynaptic spontaneous rate less than $\mu = 4.4$ Hz, satisfy $\eta < 0.25$ and the triple-wise interaction becomes negative.

Supplementary Note 5: Multicompartmental model to check the validity of theoretical boundaries of pairwise and triple-wise interactions

We simulated the membrane voltage of biologically plausible multicompartmental model neurons (https://bbp.epfl.ch/nmc-portal, L5:TTPC1:cADpyr232:1) from the data produced by the blue brain project[3,57], on the NEURON simulator (NEURON 8, Python 3.5, under Linux). The model neuron was simulation of a thick-tufted pyramidal cell in layer 5 of rat somatosensory cortex. This neuron had specific morphology and received different excitatory and inhibitory synaptic inputs from different layers (L1, L2/3, L4, L5 and L6). These input neurons activated multiple m-type excitatory and inhibitory synapses on the dendritic branches of the neuron. The m-type synaptic inputs were Poissonian events with a rate of 10 Hz. The activated input neurons are L1:DAC, L1:NGC:DA, L1:NGC:SA, L1:HAC, L23:PC, L23:MC, L23:BTC, L23:DBC, L23:LBC, L23:NBC, L23:SBC, L4:PC, L4:MC, L4:SP, L4:SS, L4:BTC, L4:DBC, L4:LBC, L4:NBC, L5:TTPC1, L5:TTPC2, L5:UTPC, L5:STPC, L5:MC, L5:BTC, L5:DBC, L5:LBC, L5:NBC, L5:SBC, L6:TPC:L1, L6:TPC:L4, L6:UTPC, L6:IPC, L6:BPC, L6:MC, L6:BTC, L6:LBC. Supplementary Fig. 7 shows how some of the input neurons activate different sites on dendritic branches of L5:TTPC1 neuron. Red sites show excitatory synapses while yellow sites show inhibitory synapses. The right bottom neuron is L5:TTPC1 neuron when all the inputs are active. In addition to these synapses, we injected presynaptic inputs conveying our shared signaling inputs with specific frequency and amplitude. The shared inputs were simple exponential synapses with a decay time constant of 0.1 ms, and applied to a 0.7 section of soma. Supplementary Table 1 shows some parameters used in simulation of multicompartmental neuron model of pyramidal neuron in L5 (https://bbp.epfl.ch/nmc-portal, L5:TTPC1:cADpyr232:1).

Parameter	Description	Value
e_{pas}	Reversal potential	-75 mV
Ra	Resistance	100 ohm-cm
g_{pass}	Membrane conductance	3e-5 S/cm^2
ena	Reversal potential for the sodium channel	50 mV
ek	Reversal potential for the potassium channel	-85 mV
ν	Inputs' rate	10 Hz
λ	Common input rate	5 Hz
syn.tau	Common input's decay time constant	0.1 ms
Δ	Time window	5 ms
dt	Simulation step size	0.025 ms
$Time$	Simulation time	300000 ms

Table 1. Some parameters used for simulation study of multicompartmental neuron using NEURON simulator. The other parameters are available in the program provided by blue brain project (https://bbp.epfl.ch/nmc-portal, L5:TTPC1:cADpyr232:1).

For the excitatory-to-trio motifs, we simulated the voltage of the pyramidal neuron (L5:TTPC1:cADpyr232:1) receiving m-type synaptic inputs in addition to Poissonian shared

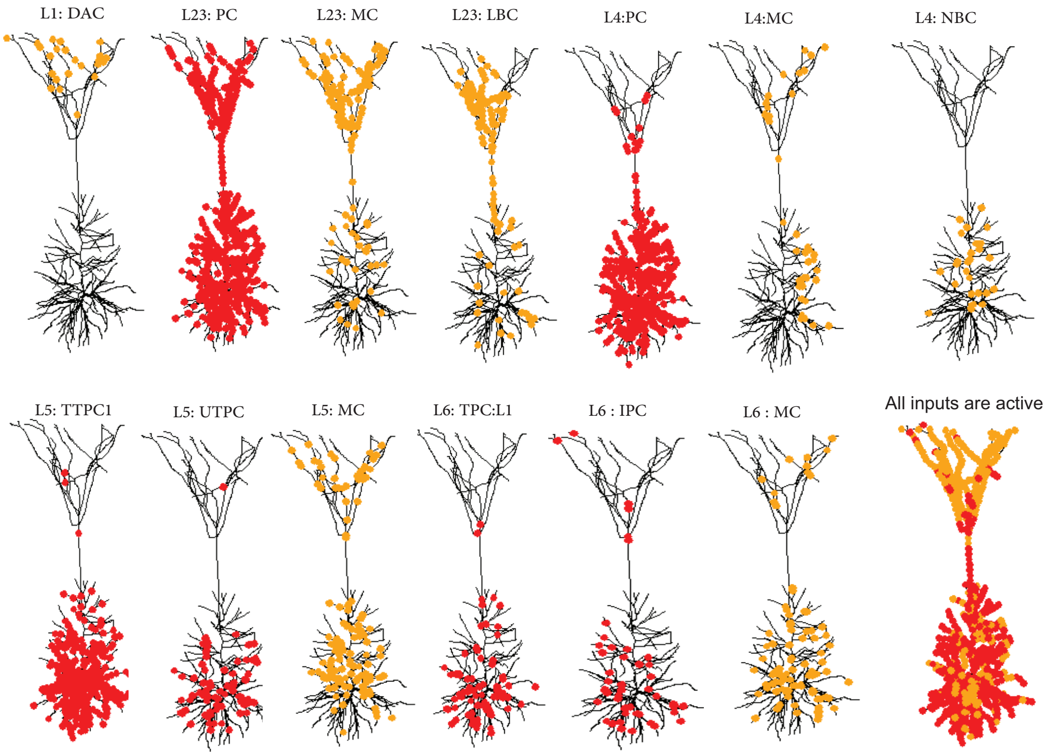

Supplementary Fig 7. Examples of synaptic activation sites from distinct presynaptic neurons on the dendritic branches of the L5 pyramidal neuron. Each panels shows the morphology of the L5 pyramidal neuron (TTPC1:cADpyr232:1). The red and orange circles are the locations of the excitatory and inhibitory synapses, respectively. The last panel shows the L5 neuron with all synaptic inputs. All the panels are generated by the NEURON program and the data provided by the blue brain project (https://bbp.epfl.ch/nmc-portal).

input with a rate of 5 Hz for 300 s (Fig. 6b, circles). The amplitude of the signaling input was within the range 0.1 to 4. We saved the spike times of the Poissonian input and used it as shared input to run simulations on two other postsynaptic neurons. To reduce the computational cost, we performed one serial session for 900 s by repeating the common input's spike times three times. Then, we calculated the interactions of these three neurons when they received one shared input on top of other synapses, and plotted them in the θ_{123} versus θ_{12} plane (Fig. 6b). We repeated the above procedure five times for Poissonian shared input with a specific amplitude. As shown in Fig. 6b, as the amplitude of shared input increases, interactions get stronger, and all the data fall within the boundaries for the excitatory-to-trio motif. We also simulated interactions among three neurons under spontaneous conditions, i.e., when they all received the m-type synaptic inputs except for the one shared input. The interactions for the spontaneous activity are nearly zero, as expected (the black diamond in Fig. 6b).

We used the same approach on the excitatory-to-pairs motif by generating three Poissonian common inputs, where each postsynaptic neuron received two of them, in addition to the m-type

synapses from different layers. We calculated the pairwise and triple-wise interactions and repeated the simulation five times for each amplitude (efficacy) of the shared signal within a range of 0.1 to 4 (Fig. 6b, squares). The simulated results are located near or in the predicted narrow excitatory-to-pairs region. For a large shared-input amplitude, the interactions reach the high-amplitude line in excitatory-to-pairs motif.

Next, we ran simulations on the inhibitory-to-trio and inhibitory-to-pairs motifs by using a multicompartmental neuron model of $L5$ in NEURON. The implementation was the same as the one for excitatory inputs, but this time, we used negative amplitudes as shared inputs and activated all other independent inputs (Poissonian with a rate of 10 Hz) the neuron received in the model of the blue brain project (L5:TTPC1:cADpyr232:1). Here, large amplitudes of inhibitory shared inputs lead to divergent results and the program encounters errors and terminates (in the blue brain NEURON program, the voltage after strong inhibitory shot goes to negative infinity), so we limited ourselves to two signaling input amplitudes (0.4 and 0.5). The result (Supplementary Fig. 8) shows that inhibitory inputs are very noisy and small, but the inhibitory-to-trio motif generates average negative triple-wise interactions (yellow circle) and the inhibitory-to-pairs motif generates average positive interactions, as predicted (yellow circle).

We also investigated the dependence of triple-wise and pairwise interactions on the simulation length. Supplementary Fig. 9 shows simulation results of pairwise (panel a) and triple-wise interactions (panel b) for the duration of 5s to 300s. They converged after about 150s (i.e. before 300s used in the above simulations). However, the duration required to obtain the desired accuracy in estimating neuronal interactions depends on the specific neuron models and synaptic inputs.

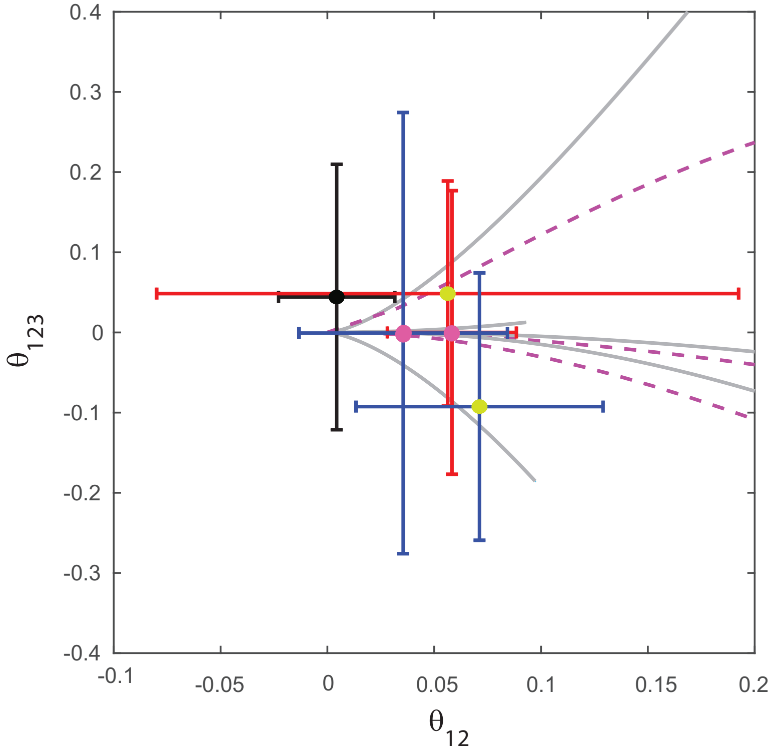

Supplementary Fig 8. Pairwise and triple-wise interactions of multicompartmental model neurons. The dot and error bars are the mean and ±SD of the pairwise and triple-wise interactions of the simulated neural activity for the inhibitory-to-trio (blue) and inhibitory-to-pairs (red) motifs using multicompartmental model neurons. The simulation was performed using NEURON for a maximum duration of 300s and repeated $5-8$ times. The amplitudes of the common inputs are 0.4 (pink circle) and 0.5 (yellow circle), and the rate is 5 Hz. The average spontaneous interactions, without shared inputs, is shown by the black circle. The time window is 5ms, and the rate of the other inputs from all the layers is 10 Hz (https://bbp.epfl.ch/nmc-portal, L5:TTPC1:cADpyr232:1).

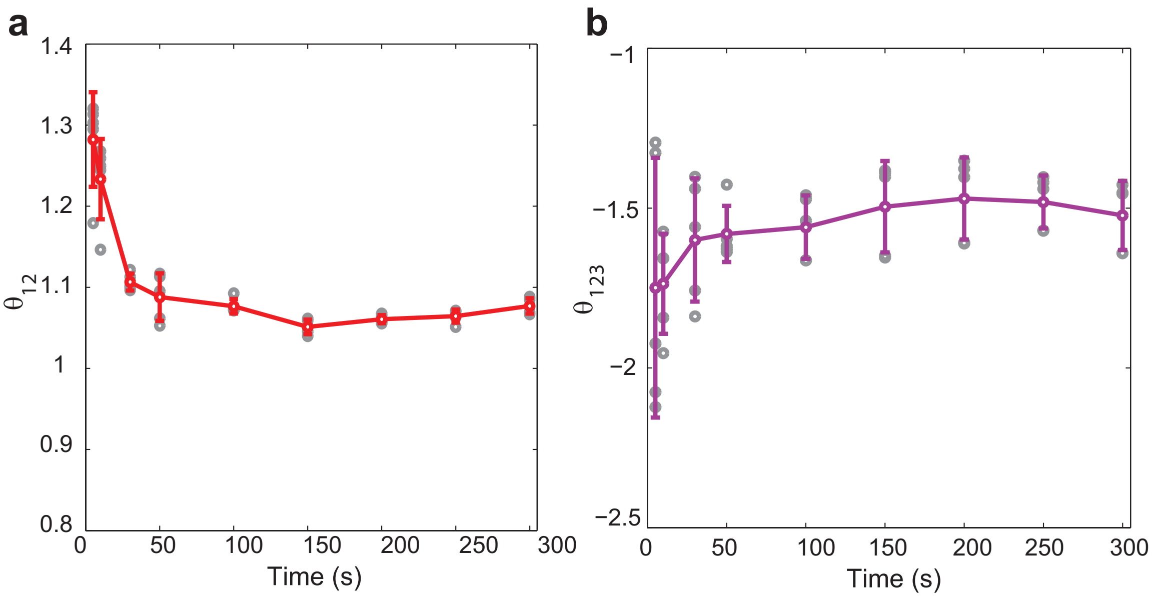

Supplementary Fig 9. Dependency of the estimated pairwise and triple-wise interactions on the recording duration for the multicompartmental neuron model. The activity of three L5 pyramidal neurons in the excitatory-to-pairs motif was simulated using the multicompartmental neuron model in the NEURON simulator environment. The simulation was done for a maximum duration of 300s and repeated 5 times. The estimated (a) pairwise and (b) triple-wise interactions are shown as a function of the data duration used for the estimation. The open circle and the error bars indicate the mean and $\pm$ SD of the 5 trials. The amplitude of the common inputs is 0.4, and the rate is 5 Hz. The time window is 5ms, and all of the other inputs have rate of 10 Hz.

Supplementary Note 6: Comparing cross-correlation with interactions of log-linear model for distinguishing the motifs

In this section, we examine the cross-correlation measure for two and three neurons to see how well it can distinguish different motifs in the pairwise (CC_2) and triple-wise (CC_3) cross-correlation plane. The cross-correlation (the Pearson correlation coefficient) is defined by normalizing the covariance between two neurons (i.e. $\langle x_1 x_2\rangle - \langle x_1\rangle\langle x_2\rangle$):

$$CC_2 = \frac{\sum_{Trial}(x_1 - \langle x_1\rangle).(x_2 - \langle x_2\rangle)}{\sqrt{\sum_{Trial}(x_1 - \langle x_1\rangle)^2 . \sum_{Trial}(x_2 - \langle x_2\rangle)^2}}, \tag{S.32}$$

where x_1 (x_2) is the activity of neuron 1 (2) and the $\langle x_1\rangle$ ($\langle x_2\rangle$) is the expectation of activity x_1 (x_2). Although the standard cross-correlation is designed to measure a linear correlation of real-valued signals, here we applied it to binary data to examine its performance in discriminating population activity patterns (i.e., x_1 and x_2 are either 0 or 1).

The cross-correlation (Eq. S.32) simplifies to:

$$CC_2 = \frac{\langle x_1 x_2\rangle - \langle x_1\rangle\langle x_2\rangle}{\sqrt{\langle (x_1 - \langle x_1\rangle)^2\rangle . \langle (x_2 - \langle x_2\rangle)^2\rangle}} \tag{S.33}$$

By representing the activity rates of neurons by probabilities of patterns, the cross-correlation can be written as[63,64]:

$$CC_2 = \frac{P(1,1) - (P(1,1) + P(1,0))(P(1,1) + P(0,1))}{\sqrt{(P(1,1) + P(1,0))(P(0,0) + P(0,1))(P(1,1) + P(0,1))(P(0,0) + P(1,0))}}, \tag{S.34}$$

where $\langle x_1 x_2\rangle = P(1,1)$, $\langle x_1\rangle = P(1,0) + P(1,1)$ and $\langle x_2\rangle = P(0,1) + P(1,1)$.

The cross-correlation among three neurons is:

$$CC_3 = \frac{\sum_{Trial}(x_1 - \langle x_1\rangle).(x_2 - \langle x_2\rangle).(x_3 - \langle x_3\rangle)}{\sqrt[3]{\sum_{Trial}(x_1 - \langle x_1\rangle)^3 . \sum_{Trial}(x_2 - \langle x_2\rangle)^3 . \sum_{Trial}(x_3 - \langle x_3\rangle)^3}}, \tag{S.35}$$

where x_1, x_2 and x_3 are the activities of neurons 1, 2 and 3, respectively. This equation can be rewritten as:

$$CC_3 = \frac{\langle x_1 x_2 x_3\rangle - \langle x_1\rangle\langle x_2 x_3\rangle - \langle x_2\rangle\langle x_1 x_3\rangle - \langle x_3\rangle\langle x_1 x_2\rangle + 2\langle x_1\rangle\langle x_2\rangle\langle x_3\rangle}{\sqrt[3]{\langle (x_1 - \langle x_1\rangle)^3\rangle . \langle (x_2 - \langle x_2\rangle)^3\rangle . \langle (x_3 - \langle x_3\rangle)^3\rangle}}, \tag{S.36}$$

Using pattern probabilities, the cross-correlation among three neurons becomes:

$$CC_3 = \frac{P(1,1,1)(1 - s - q - r) - sP(0,1,1) - qP(1,0,1) - rP(1,1,0) + 2s\,q\,r}{\sqrt[3]{(s - 3s^2 + 2s^3)(q - 3q^2 + 2q^3)(r - 3r^2 + 2r^3)}}, \tag{S.37}$$

where s, q, and r are the activity rates of neurons 1, 2 and 3.

$$\begin{aligned} s &= P(1,0,0) + P(1,1,1) + P(1,1,0) + P(1,0,1), \quad \text{(S.38a)} \\ q &= P(0,1,0) + P(1,1,1) + P(0,1,1) + P(1,1,0), \\ r &= P(0,0,1) + P(1,1,1) + P(1,0,1) + P(0,1,1), \end{aligned}$$

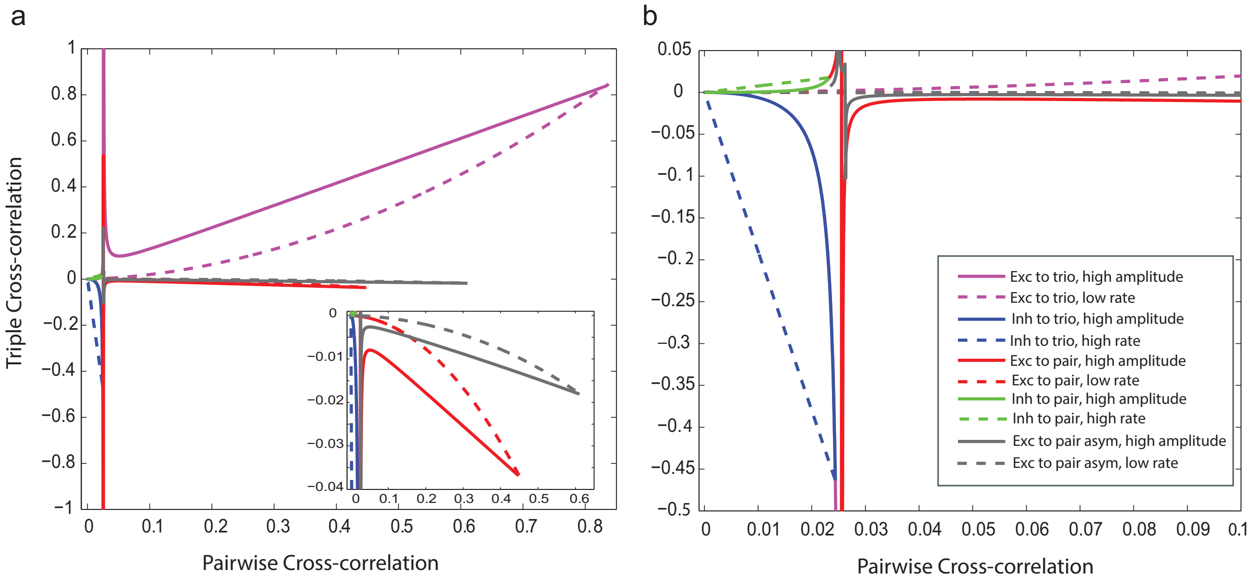

Supplementary Fig 10. Boundaries of the motifs in the triple-wise versus pairwise cross correlations plane. (**a**) One boundary arises from the high-amplitude limit of the signaling input, and the other from the low (high) spontaneous rate of postsynaptic neurons. Color codes show each motif's region that is distinguishable from the rest except for the regions where the triple-wise correlation for excitatory-to-pairs and excitatory-to-trio motifs, diverges. The divergence occurs when the denominator of CC_3 (Eq. S.39) approaches zero. The excitatory-to-trio motif induces strong CC_2 and CC_3 as the amplitude of signaling input gets stronger. The inset is a magnification of the regions for the symmetric and asymmetric excitatory-to-pairs motifs. These motifs can induce strong pairwise but small triple-wise correlations in CC_3 versus CC_2 plane. (**b**) The inhibitory-to-trio motif induces a weak pairwise but strong triple-wise correlations. The spontaneous rate ranges within $1 - 100$ Hz. Fixed parameters are $\Delta = 5$ ms and $\lambda = 5\,\text{Hz}$.

Assuming identical neurons, the cross-correlation among three neurons can be simplified as:

$$CC_3 = \frac{P(1,1,1) - 3s(P(1,1,0) + P(1,1,1)) + 2s^3}{s(s-1)(2s-1)}, \tag{S.39}$$

Using the cross-correlations CC_2 and CC_3, we drew the boundaries that define the realm of correlations achievable under different motifs like the boundaries we defined before in the plane of the information-geometric parameters (i.e., θ_{12} and θ_{123}, Fig.6a). Supplementary Fig. 10 shows the boundaries in the CC_3 versus CC_2 plane for low spontaneous rate regime (i.e., $1-100$ Hz); each motif has two boundaries: one arises from high-amplitude limit and the other from low (high) spontaneous rates for excitatory(inhibitory) inputs motifs. The motifs occupy distinct regions similar to the parameters of the log-linear model. In the cross-correlation plane, the excitatory-to-pairs motifs show strong pairwise cross-correlation but very weak triplet correlations

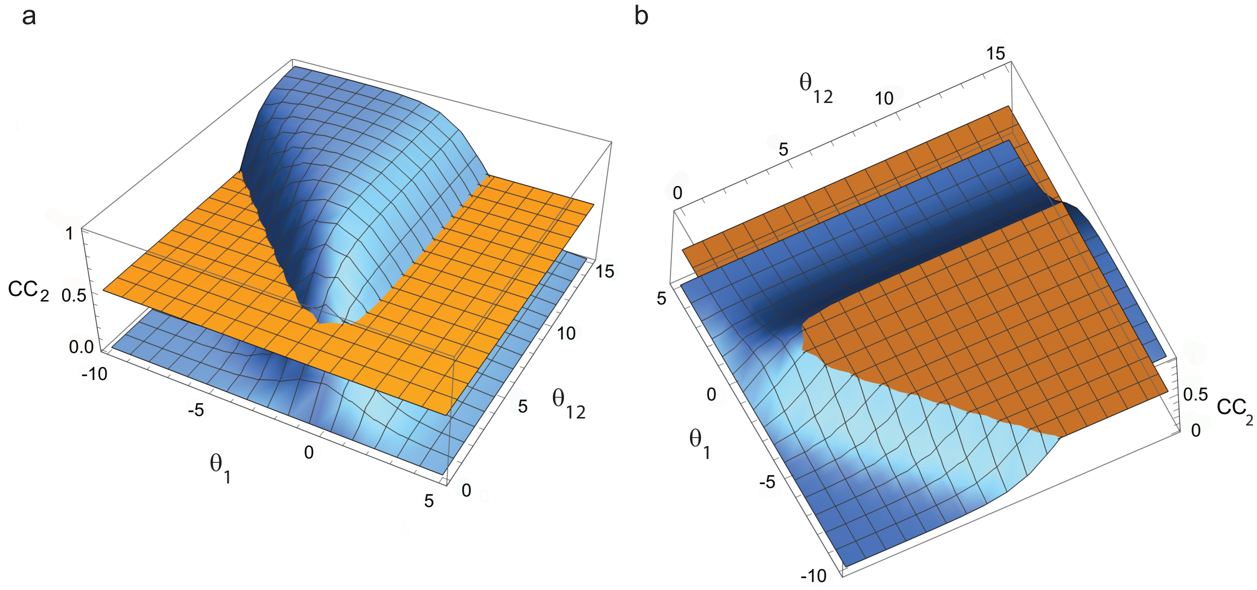

Supplementary Fig 11. Comparison of cross-correlations and interaction parameters. (**a**) The cross-correlation is shown as a function of θ_1 and θ_{12} as a blue manifold. The orange plane shows the cross-correlation of 0.5 that intersects the blue manifold. (**b**) The manifold below 0.5. The cross-correlation is 0.5 for all values of θ_{12} and θ_1 on the intersection curve.

even for large common signaling input amplitudes. In contract, inhibitory-to-trio motif shows stronger triplet but weaker pairwise correlation. Therefore, the regions are separable. However, we observed that in some range of $F_0 \approx 0.5$, the denominator of CC_3 goes to zero when $s = 0.5$ in Eq. S.39. Therefore CC_3 diverges near this point for high-amplitude boundaries of the excitatory-to-trio and excitatory-to-pairs motifs: this makes it difficult to identify the excitatory-based motifs when the spontaneous rates are near $F_0 \approx 0.5$.

If the correlation from one measure is available, it is possible to go from the parameters of this measure to another measure. For example, cross-correlation can be written in terms of the parameters of the log-linear model. By inserting pattern probabilities from Eq. 14 in Eq. S.34, the cross-correlation as a function of interaction parameter, θ_{12} becomes:

$$CC_2 = \frac{1 - e^{-\theta_{12}}}{\sqrt{(1 + e^{\theta_1})(1 + e^{-\theta_1 - \theta_{12}})(1 + e^{\theta_2})(1 + e^{-\theta_2 - \theta_{12}})}}, \tag{S.40}$$

If the two neurons are homogeneous (identical), the cross-correlation simplifies to:

$$CC_2 = \frac{1 - e^{-\theta_{12}}}{(1 + e^{\theta_1})(1 + e^{-\theta_1 - \theta_{12}})}, \tag{S.41}$$

Supplementary Fig. 11 shows how the cross-correlation depends on the natural parameters of θ_1 and θ_{12} (pairwise interaction). It is also possible to plot θ_{12} as a function of the cross-correlation. The firing rates of neuron 1 and neuron 2 are $\mu_1 = P(1,1) + P(1,0)$ and $\mu_2 = P(1,1) + P(0,1)$.

From Eq. S.34, we can determine the probability of patterns as a function of the cross-correlation. For example, $P(1,1)$ is:

$$P(1,1) = CC_2 * \sqrt{\mu_1(1-\mu_1)\mu_2(1-\mu_2)} + \mu_1\mu_2, \tag{S.42}$$

so the interaction parameter θ_{12} from Eq. 14 as a function of the cross-correlation and firing rate is:

$$\theta_{12} = \frac{1 + CC_2^2 + CC_2(\nu_1\nu_2 + (\nu_1\nu_2)^{-1})}{1 + CC_2^2 - CC_2(\nu_2/\nu_1 + \nu_1/\nu_2)}, \tag{S.43}$$

where $\nu_1 = \sqrt{\mu_1/(1-\mu_1)}$ and $\nu_2 = \sqrt{\mu_2/(1-\mu_2)}$.

When $\mu_1 = \mu_2$ ($\nu_1 = \nu_2 = \nu$), the pairwise interaction becomes:

$$\theta_{12} = \frac{1 + CC_2^2 + CC_2(\nu^2 + \nu^{-2})}{(1 - CC_2)^2}. \tag{S.44}$$

Likewise, the cross-correlation among three neurons (CC_3, Eq. S.39) as a function of the interaction parameters (Eq. 16 and Eq. 17) for homogeneous neurons becomes:

$$CC_3 = \frac{e^{3\theta_1+3\theta_{12}+\theta_{123}+2\psi} - 3e^{\psi}m(m - e^{2\theta_1+\theta_{12}} - e^{\theta_1}) + 2m^3}{m(1 + e^{2\theta_1+\theta_{12}} + 2e^{\theta_1})(1 + 2e^{\theta_1} - m)}, \tag{S.45}$$

where $m = e^{\psi}s = e^{3\theta_1+3\theta_{12}+\theta_{123}} + 2e^{2\theta_1+\theta_{12}} + e^{\theta_1}$.

Finally, the triple-wise interaction as a function of the cross-correlations (CC_3 and CC_2) for homogeneous neurons is:

$$\theta_{123} = \frac{\{(1-2\mu)\nu CC_3 + 3\mu\nu CC_2 + \mu^3\}\{(1-2\mu)\nu CC_3 - (2-3\mu)\nu CC_2 + (1-\mu)\nu\}^3}{\{1 - (1-2\mu)\nu CC_3 + 3(1-\mu)\nu CC_2 + 3\nu - \mu^3\}\{-(1-2\mu)\nu CC_3 + (1-3\mu)\nu CC_2 + \mu\nu\}^3}. \tag{S.46}$$

where $\nu = \mu(1-\mu)$ and μ is the firing rate of homogeneous neurons. Supplementary Fig. 12 compares log-linear model, cross correlation and covariance.

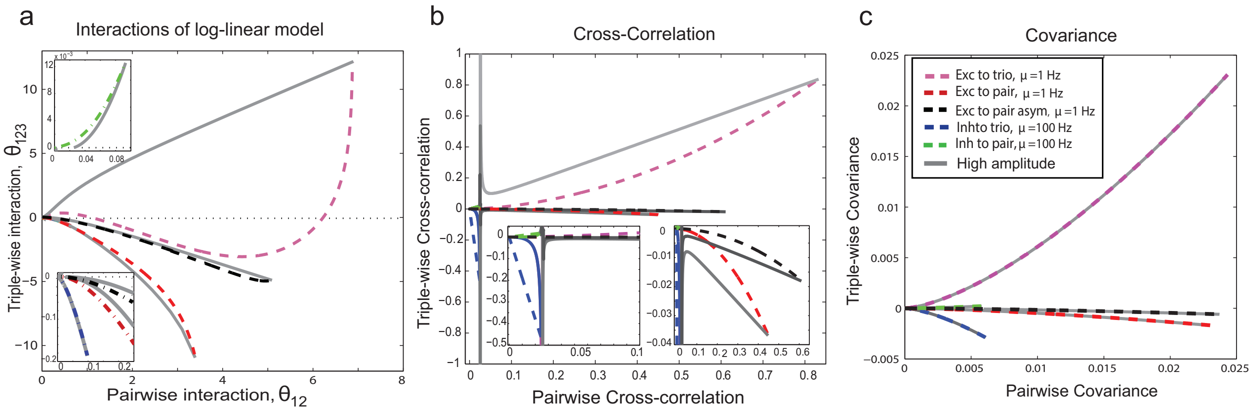

Supplementary Fig 12. Comparison of three methods of identifying motifs in the triple-wise versus pairwise correlation plane. (**a**) Interactions from the log-linear model, (**b**) cross-correlation and (**c**) covariance. The high-amplitude regime of the signaling input defines the upper boundary for each motif (gray lines). For excitatory-to-trio or excitatory-to-pairs, the low spontaneous rate ($\mu = 1$Hz) determines the other boundary (dashed purple, red, or black lines). For inhibitory-to-trio or inhibitory-to-pairs, the highest spontaneous rate ($\mu = 100$ Hz) defines the other boundary (dashed blue or green lines). Fixed parameters are $\Delta = 5$ ms and $\lambda = 5\,$Hz.

Supplementary Note 7: How does adaptation modulate the model dependent boundary?

Here we examined more physiologically plausible extensions of the LIF neuron model to see how they affect the interaction parameters and boundaries in the triple-wise versus pairwise interaction plane. In particular, we added an adaptation effect to the leaky integrate-and-fire neuron model[14,72]:

$$\tau_{\mathrm{m}} \frac{dV(t)}{dt} = -(V(t) - V_r) - w + I(t), \tag{S.47}$$

$$\tau_{\mathrm{w}} \frac{dw}{dt} = a(V(t) - V_r) - w, \tag{S.48}$$

where the adaptation current is w, the coupling term of voltage to adaptation is a, and τ_{w} is the time constant of the adaptation variable. When the neuron generates a spike, the voltage is reset to V_r and the adaptation variable w is increased by a parameter (b) for the spike-triggered adaptation, i.e., $w = w + b$. We used adaptation parameters (Supplementary Table 2) consistent with Brette and Gerstner[72]. Supplementary Fig. 13 shows the results with and without the adaptation at the threshold regime for the excitatory-to-pairs (rectangles) and inhibitory-to-trio (circles) motifs and three scaling amplitudes of signaling inputs, i.e., $A/\tau_m V_\theta = 0.05, 0.1, 0.25$. For adaptation, we considered two cases in which the coupling between voltage and adaptation current (a) is present or absent. The former (i.e. $a > 0$) considers subthreshold adaptation in addition to spike-triggered adaptation (b), while the latter (i.e. $a = 0$) assumes only the effect of spike-frequency adaptation.

Parameter	Description	Value
τ_{m}	Membrane time constant	10 ms, 20 ms
V_θ	Threshold voltage	-50 mV
V_{r}	Reset voltage	-55 mV, -70 mV
D	Diffusion coefficient	0.74 ms mV^2
τ_{w}	Adaptation time constant	144 ms
a	Subthreshold adaptation	0.0133
b	Spike triggered adaptation	2.4 mA/S
λ	Input rate	5 Hz
Δ	Time window	5 ms, 10 ms
dt	Simulation step size	0.1 ms

Table 2. Parameters used in simulation studies of extended LIF neuron model. Some parameters like the diffusion coefficient and the amplitude of shared inputs have different values and are written for each figure in the legend.

Supplementary Fig. 13 shows how adaptation modulates the pairwise and triple-wise interactions for inhibitory-to-trio (panel a) and excitatory-to-pairs motifs (panel b). Here, the adaptation makes the triple-wise (pairwise) interactions for the excitatory-to-pairs motif more negative (positive), while it makes the triple-wise (pairwise) interactions in the inhibitory-to-trio

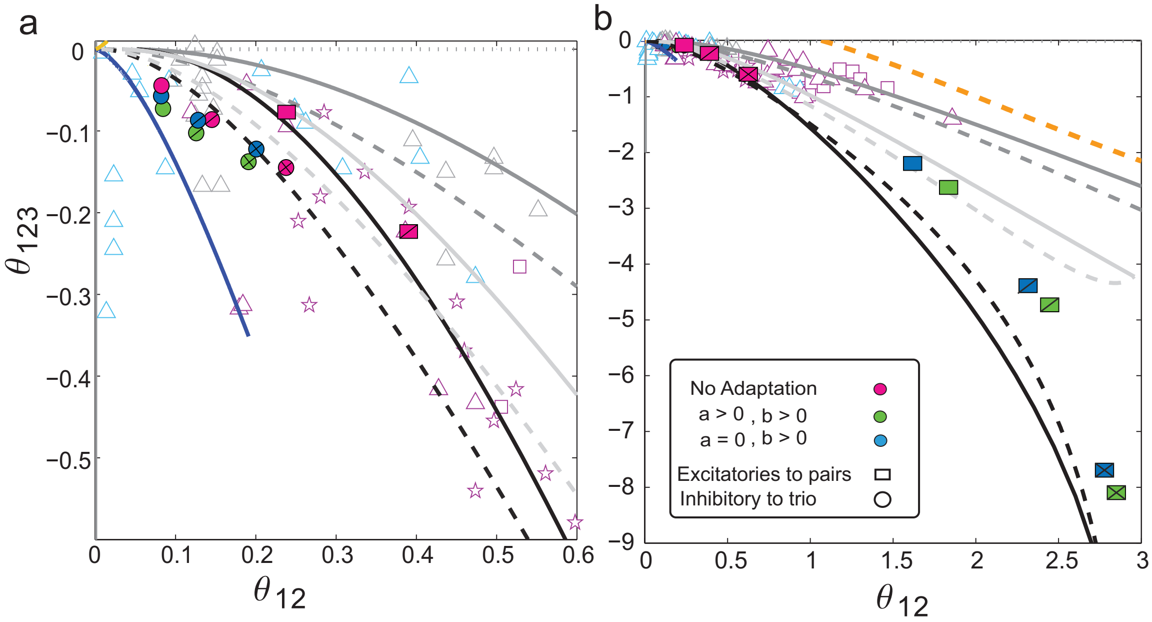

Supplementary Fig 13. Modulation of interaction parameters in the presence of adaptation. (**a**) The changes of interactions among the inhibitory-to-trio in the presence of adaptation (blue and green circles) compared with the no-adaptation case (purple circles). Each case includes results for three scaled signaling input amplitudes $A/\tau_m V_\theta$ =0.05 (no line inside the symbol), 0.1 (one line), 0.25 (two crossed lines). The interactions are stronger for larger signaling input amplitudes. (**b**) The changes of interactions among the excitatory-to-pairs motifs in the presence of adaptation (blue and green squares) compared with no adaptation case (purple squares). When adaptation is involved, the interactions are weaker for strong inhibitory-to-trios (blue and green circles) while they are stronger for excitatory-to-pairs motifs (right: blue and green rectangles). The adaptation parameters are consistent with Brette and Gerstner[72]. Fixed parameters are $a = 0.0133$ (for $a > 0$ cases), $b = 2.4\ mA/S$, $\tau_m = 10$ ms, $V_r = -70\ mV$, $V_\theta = -50\ mV$, $\tau_w = 144$ ms, $\Delta = 10$ ms, $D = 0.74\ msmV^2$, and $\lambda = 5$ Hz. Each symbol is the average of at least 10^{10} steps of the run in order to keep the mean squared error on order of 10^{-4}.

motif less negative (positive), for strong signaling inputs. This effect induces a larger difference in triple-wise and pairwise interactions between these two motifs.

The above results show adding the effect of adaptation clearly differentiates the triple-wise interactions between the excitatory-to-pairs and inhibitory-to-trio motifs in a way that the excitatory-to-pairs motifs can induce stronger negative triple-wise and positive pairwise interactions, while inhibitory-to-trio motifs induce weaker triple-wise interactions. This phenomena can be mainly attributed to decreasing the spontaneous rate of postsynaptic neurons in the presence of adaptation (Supplementary Fig. 14). We observed that adaptation changes the firing rate of the postsynaptic neuron receiving common inputs on top of noisy background inputs from 22 Hz (excitatory-to-pairs motif) and 19 Hz (inhibitory-to-trio motif) to around 5 Hz and 3 Hz respectively (see also Supplementary Fig. 15, panel b and c). When there is no common input,

adaptation decreases the firing rate of postsynaptic neurons from 22 Hz to around 3 Hz (compare number 1 blue and red in Supplementary Fig. 15, panel b). Therefore, adaptation, by reducing the spontaneous rate, increases the strength of interactions in excitatory-to-pairs motifs but decreases it in inhibitory-to-trio motifs (Supplementary Fig. 15, panel b and c).

Other interesting effects of adaptation are that excitatory-to-pairs motifs can generate the observed CV (coefficient of variation) in V1 neurons[74], and that the CV becomes high when the signaling input amplitude is increased (Supplementary Fig. 15a, solid red line). These results are consistent with experimental studies reporting high variability for neurons[16]. This effect of adaptation in increasing the variability has also been observed experimentally[73]. On the other hand, the inhibitory-to-trio motif cannot generate such a high CV (Supplementary Fig. 15a, solid blue line).

Finally, Supplementary Fig. 16 shows how adaptation modulates the model-dependent boundaries in the triple-wise versus pairwise interaction plane. Here, we used the adaptive exponential integrate-and-fire model (aEIF)[14,72] in which a term, $slop \times exp((V - V_\theta)/slop)$, is added to Eq. S.47[72]. Supplementary Fig. 16a shows how the spiking probability of a neuron within 5 ms upon signal arrival (i.e. $F_A(\Delta = 5)$) varies as a function of the scaled amplitude of signaling input for three levels of the scaled diffusion (noisy inputs) and three neuron models (LIF, aLIF, aEIF). Each plot starts from the spontaneous spiking probability ($\mu\Delta$). By increasing the scaled amplitude of the signaling inputs, $F_A(\Delta)$ gradually increases and eventually reaches 1 at high scaled amplitudes. When the scaled diffusion is 2.5×10^{-10} or less, the probability of spiking within 5 ms doesn't change for aLIF (superposition of solid red and dashed light red lines) or aEIF (superposition of solid green and dashed light green lines), so the result for $D = 2.5 \times 10^{-10}$ can be used for defining the low spontaneous rate boundaries in the θ_{123} versus θ_{12} plane. Supplementary Fig. 16b shows the boundaries of low spontaneous rate regime for excitatory-to-pairs architectures for the adaptive LIF (aLIF, Eq. S.47, red lines) and the adaptive exponential IF (aEIF, blue lines) neuron models. Each line starts at zero and, as the amplitude of common signaling inputs increases, it reaches the high amplitude boundary's curve at specific point. The point it reaches the high amplitude boundary depends on the spontaneous rate: $\mu = 1$ Hz for aLIF and 6.8 Hz for aEIF. The high-amplitude boundaries by changing the neuron model remain the same because they are independent of neuron model (gray lines). The motifs regions are still separable and can be distinguished for more biological plausible models like aLIF and aEIF.

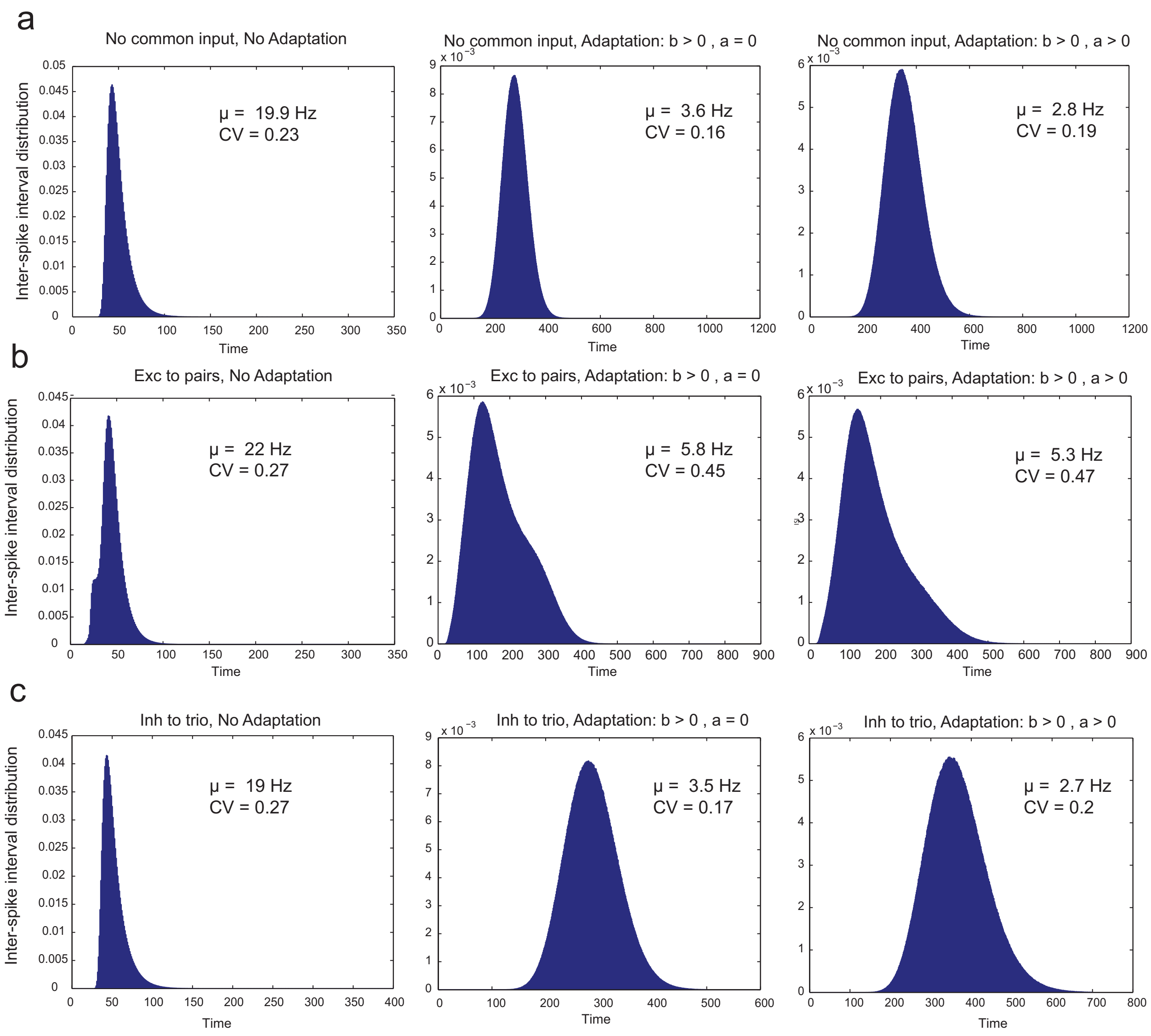

Supplementary Fig 14. Inter-spike interval (ISI) distributions of postsynaptic neurons for excitatory-to-pairs and inhibitory-to-trio motifs in model with and without adaptation. (**a**) The ISI distributions when there is no common (signaling) input without (left) or with (center, right) the adaptation. There are two conditions for adaptation ($a = 0$ or$a > 0$). In all cases, the postsynaptic neuron receives a certain level of noise that brings the mean of the membrane voltage close to the threshold. When there is no common (signaling) input, adaptation decreases the firing rate of the postsynaptic neuron, compared with no adaptation. (**b**) The ISI distributions of excitatory-to-pairs motif in model with and without adaptation. (**c**) The ISI distributions of inhibitory-to-trio motif in model with and without adaptation. For excitatory-to-pairs and inhibitory-to-trio motifs, adaptation decreases the firing rate (panel b and c, second and third row). However, adaptation increases the coefficient of variation (CV) in excitatory-to-pairs motifs and decreases the CV in inhibitory-to-trio motifs. Fixed parameters are $A/\tau_m V_\theta = 0.1$, $\Delta = 10$ ms, $D/\tau_m V_\theta^2 = 0.0002$, and $\lambda = 5$ Hz.

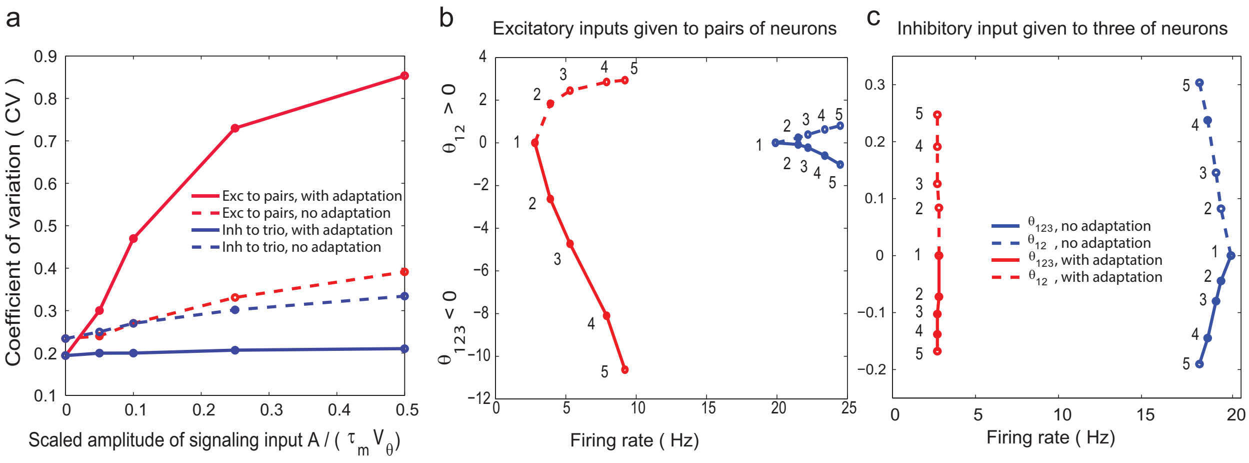

Supplementary Fig 15. Adaptation changes the coefficient of variation (CV) and interaction parameters in excitatory-to-pairs and inhibitory-to-trio motifs. (**a**) Coefficient of variation (CV) as a function of amplitude of signaling input for excitatory-to-pairs (red) and inhibitory-to-trio (blue) motifs in the presence (solid line) and absence (dashed line) of adaptation. By increasing the amplitude of excitatory-to-pairs motif, CV can reach higher values with adaptation (solid red line) than without adaptation (red dashed line). The adaptation under the inhibitory-to-trio motif decreases the CV (blue lines). (**b**, **c**) θ_{12} in the positive domain of the ordinate and θ_{123} in the negative domain as a function of the neurons' firing rate for excitatory-to-pairs (b) and inhibitory-to-trio (c) motifs. The red lines present the interactions for adaptation while the blue line is for no adaptation. The numbers $1, 2, 3, 4, 5$ indicate the amplitude of common inputs $A = 0, 10, 20, 50, 100$. The firing rate is decreased by adaptation for both excitatory-to-pairs and inhibitory-to-trio motifs (compare each number in blue and red curves) and even when there is no common input (compare number 1 in blue with red graphs). Nevertheless, θ_{12} and θ_{123} of the excitatory-to-pairs motif are increased for each amplitude of common inputs (b, compare each number in blue and red graphs). The inhibitory-to-trio motif with adaptation induces smaller interactions than in the no-adaptation case (c, compare each number's interaction in blue with red). Fixed parameters are $\Delta = 10$ ms, $D/\tau_m V_\theta^2 = 0.0002$, $\tau_m = 10$ ms, $V_\theta = 20$ mV, and $\lambda = 5$ Hz.

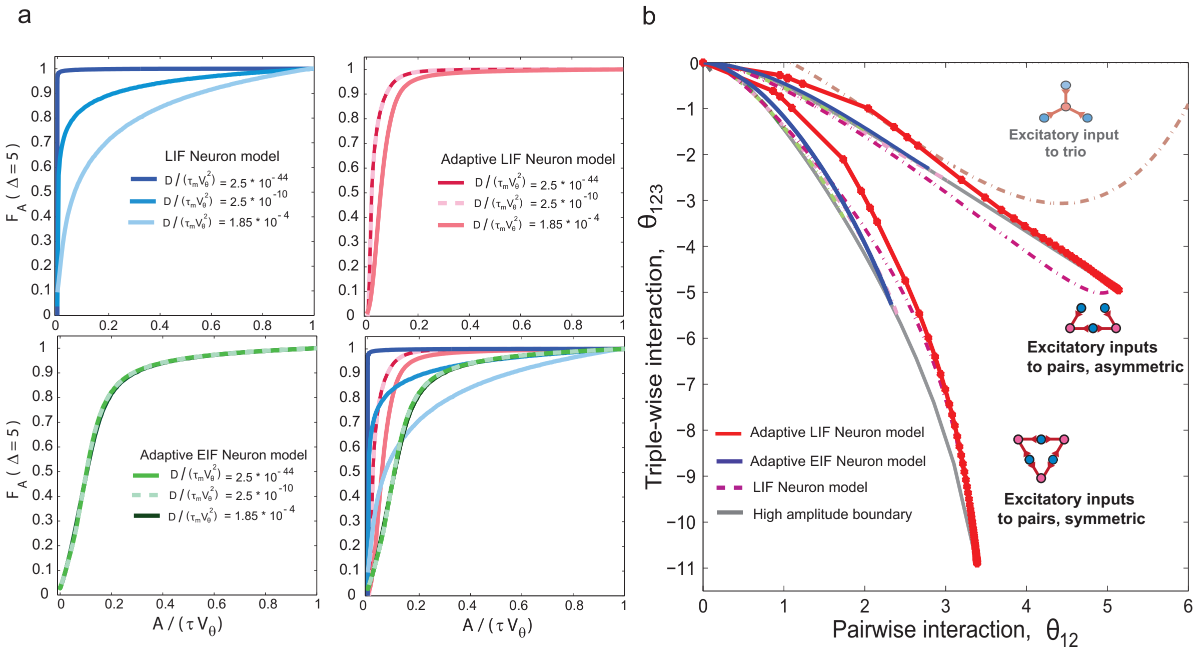

Supplementary Fig 16. Modulation of the low spontaneous rate boundaries of excitatory-to-pairs motifs by adaptive neuron models. (**a**) The probability of spiking within a time window ($\Delta = 5$ ms) after signal arrival as a function of the scaled amplitude of signaling input for different neuron models: LIF (top, left), adaptive LIF (top, right), and adaptive EIF (bottom, left). For each neuron model, the probability of spiking within 5 ms after signal arrival is shown for three levels of background noise. All the probabilities are shown together for comparison in the last graph (bottom, right). (**b**) The boundaries of excitatory-to-pairs, symmetric and asymmetric for adaptive LIF (aLIF, red) and adaptive exponential LIF (aEIF, blue) neuron model. The results for the low spontaneous rate limit are from a simulation study of aLIF and aEIF neuron models (i.e., $D/(\tau_m V_\theta^2) = 2.5 \times 10^{-10}$) and define the low spontaneous rate boundary for excitatory-to-pairs motifs, while of the high-amplitude boundary is independent of the neuron model (gray line). The brown dashed line shows the low spontaneous rate boundary for the excitatory-to-trio motif. The common parameters for the three neuron models in panel A are the membrane time constant, $\tau_m = 10$ ms, threshold voltage, and $V_\theta = 20$ mV. For adaptive neuron models, the adaptation time constant is $\tau_w = 144$ ms, coupling parameter is $a = 0.0133$, and spike-triggered average is $b = 2.4$ mV. For aEIF, the slope factor is 2 mV[72].

Supplementary Note 8: How do interactions change if we go away from the threshold regime?

Here, we investigated how the results change when we go beyond the threshold regime. We performed simulations when the postsynaptic neurons are in the subthreshold and suprathreshold regimes and compared the results with simulations at the threshold regime. Supplementary Fig. 17 shows the interactions of the simulations in the triple-wise versus pairwise interaction plane. The circles and rectangles in orange display the results when the postsynaptic neurons operate at the threshold regime. For the same parameters, those in yellow and pink are obtained when the mean inputs to the postsynaptic neurons reach 2 and 10 percent below the threshold ($\delta I/\bar{I} = -0.02, -0.1$). The circles show the interactions obtained for the inhibitory-to-trio motifs, while the rectangles are for the excitatory-to-pairs motifs. The results are for three scaled amplitudes of the signaling input at $A/\tau_m V_\theta = 0.2$ (no line inside the symbols), 0.4 (one line), 1 (two crossed lines). These results show that, as the mean input goes away from the threshold in the subthreshold regime, the interactions for the excitatory-to-pairs motifs get stronger while those for the inhibitory-to-trio get weaker.

These results can be interpreted as follows. At threshold regime, the mean input to a postsynaptic neuron is set very close to the threshold. Thus, even a small amount of noise induces spikes of the postsynaptic neuron. In subthreshold regime where the mean input given to the postsynaptic neuron is set below the threshold, the neuron generates a spike as long as the variance of the noise helps the voltage to reach the threshold. In this case, the postsynaptic neuron fires less frequently (smaller amount of $F_0(\Delta)$) compared with the case of being at the threshold regime. Since the neuron is in a regime of lower spontaneous rate, the interactions induced by the excitatory inputs increase while the interactions caused by inhibitory inputs decrease (see Supplementary Note 3). Therefore, the hypothesis that the excitatory-to-pairs motif is behind the empirically observed strong triple-wise and pairwise interactions is not only unchanged but also more strongly supported when the neuron operates in the subthreshold regime rather than operating at the threshold regime.

We then moved on to the suprathreshold regime, where the mean input is set above the threshold of the postsynaptic neuron's voltage (Supplementary Fig. 17). The symbols marked in green and blue were obtained when the mean inputs to the postsynaptic neurons reached 2 and 10 percent above the threshold ($\delta I/\bar{I} = 0.02, 0.1$). In the suprathreshold regime, the picture mentioned above reverses. Since the mean input is set above the threshold, a neuron generates regular spikes with high frequency, changing the regime of spontaneous spiking probability from low to high firing rates, $F_0(\Delta) > 0.5$. In this regime, the inhibitory signaling input can induce stronger interactions while excitatory signaling input cannot. We reconfirmed this interpretation in the simulation study (Supplementary Fig. 17). For the range of scaling amplitudes of signaling inputs $A/\tau_m V_\theta = 0.2, 0.4, 1$, we observed that the interactions induced by inhibitory inputs get stronger as the mean input increases to 2 and 10 percent above the threshold (compare the orange circles with the green and blue circles of the symbols with crossed lines, for example).

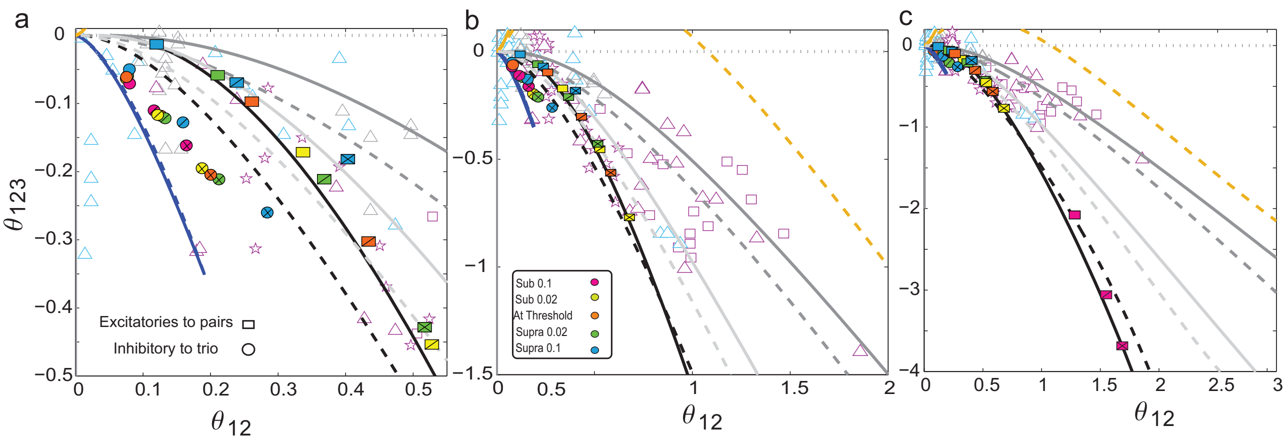

Supplementary Fig 17. Modulation of interaction parameters in subthreshold and suprathreshold regimes where mean synaptic input given to membrane potential is set below or above the threshold. (**a**) Changes in interactions among inhibitory-to-trio motifs in subthreshold and suprathreshold regimes. Each color represents deviations from the threshold regime (see the color code) at three scaled amplitudes of the signaling input at $A/\tau_m V_\theta = 0.2$ (no line inside the symbols), 0.4 (one line), 1 (two crossed lines). (**b**, **c**) The interactions for the motif of excitatory-to-pairs of neurons. When a neuron is in the subthreshold regime, the interactions are reduced for inhibitory-to-trio motif (compare the orange with the pink and yellow circles) while they are increased for those of excitatory-to-pairs motif (compare orange with pink and yellow rectangles). In the suprathreshold regime, the excitatory-to-pairs motif generates weaker interactions (green and blue rectangles), while the inhibitory-to-trio motif generates stronger interactions, especially for stronger input (green and blue circles with crossed lines inside). Fixed parameters are $\Delta = 10$ ms, $D/\tau_m V_\theta^2 = 0.002$, and $\lambda = 5$ Hz. Each symbol is the average of at least 10^{10} steps of the run so as to keep the mean squared error on the order of 10^{-3}-10^{-4}.

Supplementary Note 9: Does mixing motifs, excluding the excitatory-to-pairs motif, generate strong negative triple-wise interactions?

We have four architectures, common inputs given to pairs or to trio of postsynaptic neurons with either common excitatory or inhibitory inputs. Based on our analysis in this paper on common inputs and also recurrent activity, we know that the excitatory-to-pairs motif, if it exists, can induce strong interactions of $\theta_{12} > 0$ and $\theta_{123} < 0$. However, we can ask the following question: by mixing the other motifs (i.e, inhibitory-to-trio, excitatory-to-trio and inhibitory-to-pairs), can we reach a large magnitude of $\theta_{12}(> 0)$ and $\theta_{123}(< 0)$? For this purpose, we mixed the three motifs, two by two, to see whether such a case exists. We mixed two motifs with firing rates of $\lambda_1 = 5$ Hz and $\lambda_2 = 5$ Hz for a range of common input amplitudes in each motif (i.e. from $A = 0$ to $A = 50$). The results of mixing the excitatory-to-trio motif with the inhibitory-to-trio motif are shown in Supplementary Fig. 18a. Although the inhibitory-to-trio motif induces negative triple-wise interactions, the interactions are mostly positive when it is mixed with the excitatory-to-trio motif. The only region in which the mixing triple-wise interactions are negative is the weak or near-zero amplitudes of excitatory-to-trio motif (Supplementary Fig. 18a). The mixture of excitatory-to-trio and inhibitory-to-pairs motifs induces positive triple-wise interactions (Supplementary Fig. 18b) as expected from its each individual motif. The results for mixing the inhibitory-to-trio and inhibitory-to-pairs are shown in Supplementary Fig. 18c. Although the inhibitory-to-pairs motif induces positive triple-wise interactions, when it is mixed with the inhibitory-to-trio motif, negative triple-wise interactions occur for most amplitudes of mixing (Supplementary Fig. 18c). The interactions induced in this mixing are in the range of interaction of the inhibitory-to-trio motif, which is not strong and does not interfere with the excitatory-to-pair motif's range of interaction. The triple-wise and pairwise interactions in each case, saturate at a specific amplitude of common input.

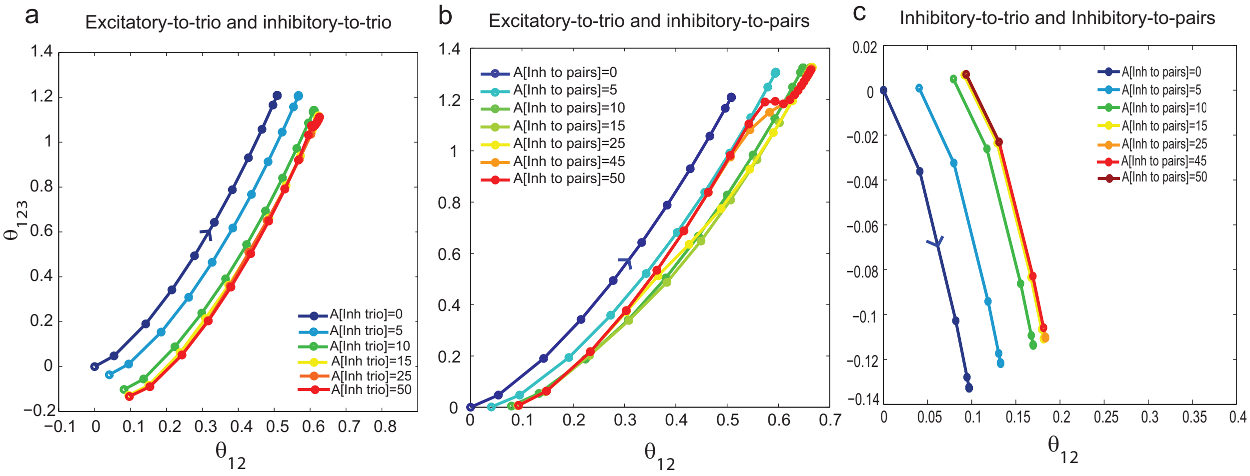

Supplementary Fig 18. Effect of mixing the motifs of excitatory-to-trio, inhibitory-to-trio, and inhibitory-to-pairs. The results of interactions are shown for (**a**) the mixtures of excitatory-to-trio and inhibitory-to-trio, (**b**) excitatory-to-trio and inhibitory-to-pairs, and (**c**) inhibitory-to-trio with inhibitory-to-pairs (right). The amplitude of common input in each motif varied from 0 (i.e., the motif is inactive) to 50 (arrow shows increasing amplitude in each panel), and the interactions (θ_{123} and θ_{12}) were calculated respectively. Along each colored line (following the arrow in each panel), one motif's amplitude is fixed while the other mixed motif's amplitude varies from 0 to 50. For example, in panel **a**, for each colored line, the amplitude of the inhibitory-to-trio motif is fixed (see color box), while the amplitude of the excitatory-to-trio motif changes along the line from 0 to 50. The color box shows in each panel which motif has the fixed amplitude. Fixed parameters are $\Delta = 10$ ms, $D/\tau_m V_\theta^2 = 0.003$, $\tau_m = 10$ ms, $V_\theta = 5$ mV, $\lambda_1 = 5\,\text{Hz}$ and $\lambda_2 = 5\,\text{Hz}$.

Supplementary Note 10: How geometrical constraints modify the probability of finding various microcircuits: A simple Monte Carlo approach

We wrote simple MATLAB code using a 10^8 times uniform random number generator. It puts points on the XY-plane, in a $2r \times 2r$ square, with $r = 150\,\mu$m. Then, excluding any point further from the origin (i.e. at $x = 0$ and $y = 0$) than r, we simply have a pool of random points, all sitting in a circle of radius r= $150\,\mu$m. By picking each pair (or trio), we can verify if two (or three) picked points are closer to each other than the critical length of $l_c = 100\,\mu$m. This simply produces two populations among all possible choices of two (or three) points. Out of 4.8×10^7 choices of pairs, we found that the probability of the two points being closer to each other than $l_c = 100\,\mu$m was 0.32, and the probability of finding three points, each point closer to two others than $100\,\mu$m, was 0.07.

Supplementary References

1. Amari, S.-i. Measure of correlation orthogonal to change in firing rate. *Neural computation* **21**, 960–972 (2009).

2. Martignon, L. *et al.* Neural coding: higher-order temporal patterns in the neurostatistics of cell assemblies. *Neural Computation* **12**, 2621–2653 (2000).

3. Nakahara, H. & Amari, S.-i. Information-geometric measure for neural spikes. *Neural Computation* **14**, 2269–2316 (2002).

4. Shlens, J. *et al.* The structure of multi-neuron firing patterns in primate retina. *Journal of Neuroscience* **26**, 8254–8266 (2006).

5. Staude, B., Rotter, S. & Grün, S. Cubic: cumulant based inference of higher-order correlations in massively parallel spike trains. *Journal of computational neuroscience* **29**, 327–350 (2010).

6. Ohiorhenuan, I. E. & Victor, J. D. Information-geometric measure of 3-neuron firing patterns characterizes scale-dependence in cortical networks. *Journal of computational neuroscience* **30**, 125–141 (2011).

7. Brunel, N. & Hakim, V. Fast global oscillations in networks of integrate-and-fire neurons with low firing rates. *Neural computation* **11**, 1621 (1999).

8. Brunel, N. Dynamics of sparsely connected networks of excitatory and inhibitory spiking neurons. *Journal of computational neuroscience* **8**, 183–208 (2000).

9. Shomali, S. R., Ahmadabadi, M. N., Shimazaki, H. & Rasuli, S. N. How does transient signaling input affect the spike timing of postsynaptic neuron near the threshold regime: an analytical study. *Journal of computational neuroscience* **44**, 147–171 (2018).

10. Markram, H. *et al.* Reconstruction and simulation of neocortical microcircuitry. *Cell* **163**, 456–492 (2015).

11. Ramaswamy, S. *et al.* The neocortical microcircuit collaboration portal: a resource for rat somatosensory cortex. *Frontiers in neural circuits* **9**, 44 (2015).

12. Tatsuno, M. & Okada, M. Investigation of possible neural architectures underlying information-geometric measures. *Neural computation* **16**, 737–765 (2004).

13. Brette, R. & Gerstner, W. Adaptive exponential integrate-and-fire model as an effective description of neuronal activity. *Journal of neurophysiology* **94**, 3637–3642 (2005).

14. Gerstner, W., Werner, M. K., Naud, R. & Paninski, L. *Neuronal dynamics: From single neurons to networks and models of cognition* (Cambridge University Press, 2014).

15. Gur, M., Beylin, A. & Snodderly, D. M. Response variability of neurons in primary visual cortex (v1) of alert monkeys. *Journal of Neuroscience* **17**, 2914–2920 (1997).

16. Shadlen, M. N. & Newsome, W. T. The variable discharge of cortical neurons: implications for connectivity, computation, and information coding. *The Journal of neuroscience* **18**, 3870–3896 (1998).

17. Adibi, M., McDonald, J. S., Clifford, C. W. & Arabzadeh, E. Adaptation improves neural coding efficiency despite increasing correlations in variability. *Journal of Neuroscience* **33**, 2108–2120 (2013).
